# Bacteriophage Density Influences the Rate of Resistance Evolution

**DOI:** 10.1007/s11538-026-01704-5

**Published:** 2026-08-20

**Authors:** Tuan Phan, Anuja Shrestha, Jacob Schow, Craig R. Miller, Tracey L. Peters, James T. Van Leuven

**Affiliations:** 1 Department of Mathematics, Augusta University, Augusta, USA; 2 Institute for Modeling Collaboration and Innovation, University of Idaho, Moscow, USA; 3 Department of Animal Veterinary and Food Sciences, University of Idaho, Moscow, USA; 4 Department of Biological Sciences, University of Idaho, Moscow, USA

**Keywords:** Bacteriophage resistance evolution, Multiplicity of infection, Generalized Lotka-Volterra (gLV), Ordinary differential equations, Competitive dynamics, Fitness costs of resistance, Phage-density-dependent competition, Profile likelihood

## Abstract

The antagonistic relationship between bacteria and bacteriophages (phages) drives genetic changes that result in phage resistance, making it important to understand the dynamics of resistance acquisition in both natural microbial communities and therapeutic contexts. We developed a generalized Lotka–Volterra model describing the interaction among phage-susceptible bacteria, phage-resistant bacteria, and a lytic phage, incorporating asymmetric interspecific competition and partial phage resistance. The model was fitted to growth data from four bacterial strains—*Pseudomonas aeruginosa* PA103 and PAK challenged with a phage cocktail, and *Paenibacillus larvae* Y-3650 and 25747 challenged with phage Fern IDv1—across four levels of multiplicity of infection (MOI). We compared two parameterization schemes: M1, in which the interspecific competition coefficients $a_{rs}$ and $a_{sr}$ are shared across MOI treatments, and M2, in which these coefficients are allowed to vary by treatment. For the two *P. aeruginosa* strains, M1 produced adequate fits (mean $R^2 = 0.91$ and 0.89). For the two *P. larvae* strains, M1 failed catastrophically at specific treatments ($R^2$ as low as -19.7), while M2 achieved mean $R^2$ of 0.90 and 0.95 for Y-3650 and 25747, respectively. Information-theoretic model comparison ($\Delta \text {AIC} = -753$ and -458) and profile likelihood analysis confirmed that the competition coefficients are fundamentally MOI-dependent, with per-treatment optima diverging by up to three orders of magnitude. These findings indicate an effect that extends beyond phage-mediated apparent competition—which assumes zero direct competition between the susceptible and resistant subpopulations—because the fitted interspecific competition coefficients are nonzero and MOI-dependent. We observed three distinct growth patterns—delayed growth, two-phase growth, and complete suppression—with non-monotonic dose–response relationships in which intermediate phage doses produced stronger suppression than higher doses. This non-monotonicity demonstrates that the boundaries between growth regimes are governed by competitive dynamics rather than phage dose alone. Our results establish that phage resistance evolution is fundamentally an ecological process: the competitive context, shaped by phage pressure, determines population-level outcomes independently of intrinsic mutation capacity. These findings have implications for phage therapy, suggesting that optimal dosing strategies must account for the competitive environment created by treatment, not solely the direct bactericidal effect.

## Introduction

Generalized Lotka-Volterra (gLV) ordinary differential equations (ODEs) are commonly used to describe bacteria-phage dynamics (Lenski 1988; Levin et al. 1977; Campbell 1961). These ODEs have been modified to accommodate various properties of microbial communities (e.g., carrying capacity, lag in phage growth, phage and bacterial evolution, etc.). The summation of decades of work on this subject is a generally accepted theoretical framework that is mostly consistent with empirical studies (Lenski 1988; Levin and Bull 2004; Styles et al. 2021). Co-cultured phages and hosts undergo an initial period of dynamics where logistic growth of the host is followed by growth of phage and a crash in host density. The host population can recover and achieve a steady-state (stable coexistence of phage and hosts) when susceptible hosts are at low enough density that they escape phage predation or when phage-resistant variants arise and induce a reciprocal cycle of co-evolution between phage and bacteria. Costs associated with the adaptation of phage resistance may also be invoked to explain why resistant hosts do not entirely displace susceptible hosts (Lenski 1988; Bohannan et al. 2002).

A foundational contribution to the theoretical understanding of phage therapy was made by Payne and Jansen (2001), who analyzed phage therapy as a density-dependent kinetic process. Their model identified key thresholds—including a proliferation threshold below which phage cannot amplify sufficiently to clear an infection—and predicted several qualitatively distinct outcomes depending on the initial ratio of phage to bacteria: successful clearance, failed therapy, and intermediate regimes of partial suppression. Subsequent work extended this framework to pharmacokinetic principles of phage dosing (Payne and Jansen 2003), quantitative in vitro models incorporating resistant bacteria and latent periods (Cairns et al. 2009), and the concept of a phage replication threshold (Kasman et al. 2002). Together, these studies established that the outcome of phage therapy depends critically on the initial densities of phage and bacteria, the kinetics of phage amplification, and the emergence of resistance. However, most theoretical treatments in this tradition have focused on the direct interaction between phage and a single bacterial population, without explicitly modeling the competitive dynamics between susceptible and resistant subpopulations. How the competitive environment between bacterial subpopulations changes as a function of phage dose—and whether this competitive context, rather than mutation rate alone, governs resistance evolution outcomes—remains an open question.

Several intriguing inconsistencies remain between this standard model and empirical results. One pertains to the initial phage inoculum size. Kysela (2007) developed an ODE model to investigate the role of phage mutation rates on the outcome of phage therapy (Kysela and Turner 2007). They found that, contrary to expectation, the peak density of phage-resistant bacteria increases with the multiplicity of infection (MOI) up to about 10-100 phage/host cell, at which point it decreases. The authors suggest that a high MOI decreases the susceptible host density and thus limits the emergence of a second class of phages, which can infect phage-resistant bacteria. This limitation on the phage growth allows the total bacterial density to be higher than if the MOI were lower than around 10. Exploring the impact of phage density on resistance evolution is important because the gut microbiome of most animals is known to be dynamic (Kešnerová et al. 2019; Bobay et al. 2020; Gibbons et al. 2017; Priya and Blekhman 2019) yet many previous empirical studies explore a fairly narrow range of host and phage densities. Moreover, the pharmacokinetics of phage therapy treatments is still poorly defined, thus it is likely that a wide range of phage and host densities are being unwittingly used. It is imperative to understand the importance of these parameters in the outcome of resistance evolution.

We were initially motivated to model resistance evolution to better understand resistance arising during the Appelmans protocol (Burrowes et al. 2019), a method used to expand the host range of a phage cocktail through serial passage on multiple hosts. While performing Appelmans in microtiter plates with optical density measured every 10 minutes, we frequently observed two-phase growth: an initial increase in cell density, followed by a phage-induced decline, and then a second rise to stationary phase-consistent with the emergence of a phage-resistant subpopulation. However, because the Appelmans protocol includes a series of ten-fold phage dilutions, we could track these dynamics across a wide range of phage doses. A recurring and unexpected pattern emerged: at higher phage concentrations, the resistant population often rose more rapidly than at lower concentrations. Our initial expectation was that a standard model of phage and host replication kinetics would account for this pattern, but simulations parameterized from experimental growth data did not reproduce the observed dose-dependent acceleration of resistance. This discrepancy suggested that the competitive interaction between susceptible and resistant bacteria—not just phage lysis kinetics–changes as a function of phage pressure.

To test this hypothesis, we developed a generalized Lotka–Volterra model that explicitly incorporates asymmetric competition between susceptible and resistant bacterial subpopulations, partial phage resistance, and resistance acquisition. We fitted this model to experimental growth data from two *Pseudomonas aeruginosa* and two *Paenibacillus larvae* strains across multiple MOI levels, and used profile likelihood analysis and information-theoretic model comparison to test whether the interspecific competition coefficients are MOI-dependent. Our results demonstrate that phage pressure reshapes the competitive landscape between susceptible and resistant bacteria, and that resistance evolution is governed by an ecological process in which competitive context determines outcomes independently of mutation capacity.

## Methods

### Bacterial strains and phages

The strains of *Paenibacillus larvae* from ERIC group I were used in the study. Strain Y-3650 was acquired from The University of Nevada Las Vegas (Yost et al. 2016), whereas the other strains: NRRL B-2605 and ATCC-25747 were received from the American Type Culture Collection (ATCC).

The phage strains specific to *Paenibacillus: *Fern, Xenia, and Scottie were also acquired from the University of Nevada Las Vegas (Stamereilers et al. 2016). Phages Fern (KT361649) and Xenia (KT361652) were isolated from infected larvae (Tsourkas et al. 2015) whereas phage Scottie (MH460825) was isolated from commercial hand cream purchased in the Las Vegas area (Yost et al. 2018). These isolates were resequenced when we acquired them. Genome differences were described in Spencer et al. (2025) and the phages were renamed Fern_IDv1, Xeni_IDv1, and Scot_IDv1 to conform with International Committee on the Taxonomy of Viruses (ICTV) standards and differentiate them from the originally published phages which were quite diverged in nucleotide sequence.

All *P. aeruginosa* bacterial strains and their hosts were previously described in Peters et al. (2024). Strains PAK wt Lee, PA103 wt, and PDO300 wt Parsek were received from Dr. Pradeep Sing at the University of Iowa in 2006. PAO1 wt and PA14 wt were a gift from Dr. Jim J. Bull at the University of Idaho, originally given to him from Dr. Joe A. Fralick at Texas Tech University.

*P. aeruginosa* phages included M6, F116, and JM2. M6 was received from Denise Tremblay from the University of Laval Oral Ecology Research Group in 2006. Phages M6 and F116 are commonly used laboratory strains. JM2 was isolated by Jack Milstein at the University of Idaho in 2006 from a Moscow, ID sewer sample. All bacteria and phages were stored at $-80^{\circ }$C in 750 μL of LB media with 30% glycerol. See Peters et al. (2024) for a complete description of these phages.

### Bacteria and phage propagation

*P. larvae* bacterial strains were streaked from their respective -$80^\circ$C glycerol stocks onto 1 mg/L of thiamine hydrochloride Brain Heart Infusion (BHI) agar (Catalog#DF0418-17-7, BD Difco^TM^, USA) plates and incubated at $37^\circ$C with 5% CO_2_ for 72 hours. A single clean colony from each streaked plate was grown in 1 mg/L of thiamine hydrochloride BHI broth (Catalog#CM1135B, Thermo Scientific^TM^, USA) at $37^\circ$C in a shaking incubator at 120 rpm (3 replicates/bacterial strain). After 24 hours of incubation, the bacterial cultures were used for assays. The same bacterial cultures were used to count CFU/mL.

The same basic cell culture methods were used for *P. aeruginosa* except that the propagation media used was Lysogeny broth (LB) and LB-agar. LB is composed of 10 g/L tryptone, 5 g/L yeast extract, and 10 g/L NaCl. Agar was used at a 1.5% with a 0.7% top-agar overlay. *P. aeruginosa* was cultured at $37^\circ$C under atmospheric conditions for 14-16 hours.

To resurrect phages from -80 stocks, a loopful of each phage freezer stock was suspended in 200 μL of broth and mixed thoroughly. This stock was serially diluted and titered on *P. larvae* bacterial strain Y-3650, a host permissive to all *P. larvae* phages used in this study. A clean plaque from the Y-3650 titer plate was picked using a sterile cut pipette tip and deposited in a microcentrifuge tube with 750 μL of BHI broth. After gently mixing, 50 μL of chloroform was added to the tube, vortexed thoroughly followed by centrifugation at 15,000 x *g* for 5 minutes. 500 μL of the supernatant was collected and stored in a new tube. This phage stock was then used to make a high titer working phage stock. For this, a 1 mL overnight culture of Y-3650 was prepared from a streaked plate. The 1 mL overnight culture was used to inoculate 9mL of BHI broth. The positive control was a flask of overnight Y-3650 culture: BHI broth (1:10) and the negative control was a flask with 10 mL of BHI broth. Cell growth was measured using optical density (OD) with wavelength of 600 nm. Once the OD 600 reached 0.2 (at around 3 hours), 100 μL of the phage stock was added to the flask. These flasks were incubated in a shaker incubator for 24 hours at 200 rpm. The OD 600 measurement can be affected by cell clumping, which causes stochastic values as the light beam cannot easily pass though large cell clumps. This seems to have affected our measurements of *P. larvae Y-3650* as seen in Figure 2D. The lysed bacterial cells and spent culture media were aliquoted into 1.5 mL tubes (1 mL/tube). 50 μL of chloroform was added to each tube, vortexed thoroughly and centrifuged at 15,000 x *g* rpm for 5 minutes to fully separate the aqueous layer from the chloroform and any other cell debris. The supernatant was extracted and used as the working phage stock.

Phages were titered by soft-agar overlay. Briefly, a serial dilution of each phage strain was prepared using liquid broth. 3 mL of nutrient-rich top agar (0.7%) enriched with 1mM CaCl_2_ and 1 mM MgCl_2_ was mixed with 100 μL of each host strain and 100 μL of each diluted phage stock, mixed well by briefly vortexing, and poured on the top of a 1.5% agar plate. The use of CaCl_2_ and MgCl_2_ helps in facilitating phage attachment to their host Yost et al. (2016). Plates were then incubated with their lids facing down, at $37^\circ$C for 24 hours. The plaques on the plates were counted and the phage stock titers were calculated based on the number of plaques present on the plate and the phage dilutions stock used for plating.

### Host range determination

Spot test assays were conducted to determine whether the *P. larvae* specific phages were able to infect the *P. larvae* strains. For this, gridded lines/boxes were made on BHI agar plates and each box was labeled with each phage titration. Then, 100 μL of overnight *P. larvae* strain culture was added to a 3 mL of BHI top agar enriched with 1 mM CaCl_2_ (Catalog#CAS 10035-04-8, Fisher Scientific^TM^, USA) and 1 mM MgCl_2_ (Catalog#CAS 7791-18-6, Fisher Scientific, USA) and poured on the labeled BHI agar plate and let it sit down for ∼ 30 minutes. Then, 10 μL of serially diluted phage stock (10^0^ to 10^-9^) was spotted onto each labeled gridded box. The plates were left to sit down for about an hour to avoid spreading the phage droplets, followed by incubating plates with lids upright at $37^\circ$C with 5% CO_2_ for 24 hours and lysis of each phage activity to its host was noted. If plaques were observed on the plates, then, phage titration plates were made as described below. No plaques on the plates mean that specific *P. larvae *phage failed to lyse that specific *P. larvae* strain on the lawn of which the spots were made. Here, *P. larvae* phage Xenia_IDv1 failed to lyse *P. larvae* strain ATCC-25747.

### Appelmans protocol

A phage stock was prepared by propagating a single plaque on a permissive host overnight. The resulting lysate was treated with 10% chloroform, then centrifuged at 15,000 x *g* for 5 minutes. 500 μL of the supernatant was collected and stored in a new tube. This phage stock was stored at $4^\circ$C during the experiment.

Overnight cultures of *P. larvae* strains were used for the assays. Each assay plate had a total of 8 treatments with 3 replicates per treatment. Of the 8 treatments, 3 were controls: BHI broth control, bacteria control, and phage control. The BHI control (only growth media) provides a background absorbance level to normalize to and ensures the media is not contaminated. The bacteria controls consist of bacteria with media but without phage at the same concentrations as the treatment samples. This provides growth rates for each bacterial strain. The phage control consists of phage with media but without host bacteria. This ensures that the phage samples are not contaminated with any bacteria. The remaining 5 were the treatments with different phage titers with the same volume and same concentration of bacterial strain. Each assay plate was incubated in a BioTek microplate reader (BioTek^®^ Instrument, Inc. USA) with shaking for 48 hours with the reading of optical density (OD_600_) recorded from each well at the intervals of every 15 minutes.

Overnight cultures of *P. aeruginosa* strains were normalized to an OD 600 nm of 0.3, then diluted 1:10. 1 μL of the diluted hosts was pipetted into each well (a total of roughly 1000 cells per well). Starting with phage stock at a concentration of 1E7 PFU/mL, a dilution series was made from 1E0 to 1E9 dilution and 100 μL of these diluted phages were pipetted into each well. The final volume of the culture was 200 μL per well in 96 well plates. Thus, the maximum resulting MOI was around 1000 PFU/CFU and the minimum MOI was around 1E-6 PFU/CFU.

Cell densities of *P. larvae* and *P. aeruginosa* were measured by counting colonies on agar plates. 2 mL of mBHI was in 24 well plates was inoculated with *P. larvae* Y-3650 at a density of approximately 4.2E6 CFU/mL and 25747 was at a density of 2.4E6 CFU/mL. Phages Fern_IDv1, Xeni_IDv1, and Scot_IDv1 were introduced at a maximum concentration of 2.0E2 PFU/mL, 5.6E2 PFU/mL, and 4.3E2 PFU/mL, respectively.

### Mathematical model setup

**Fig. 1:**
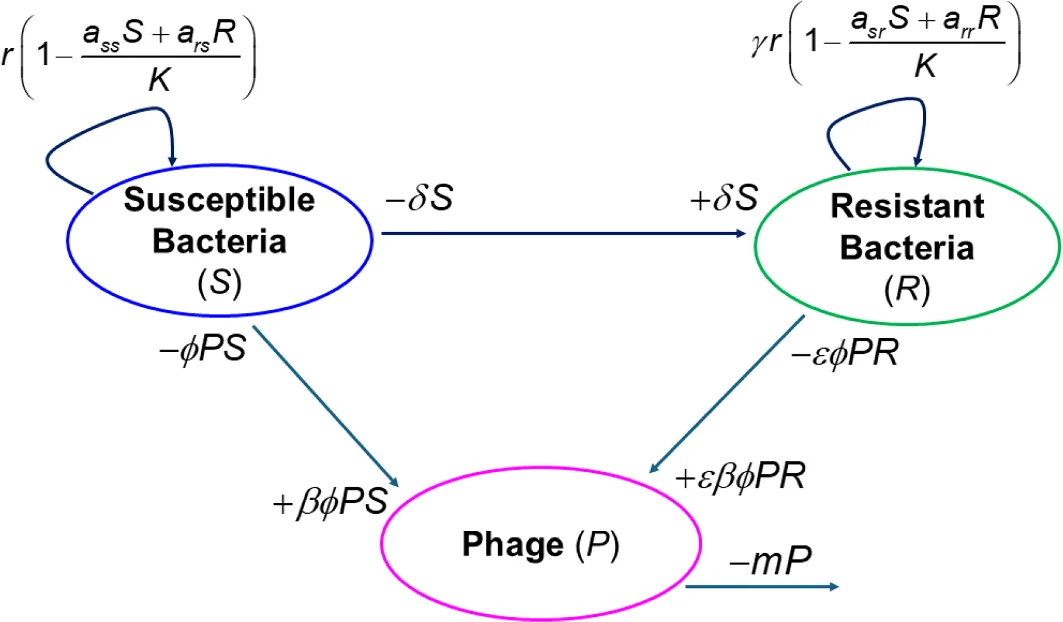
Schematic of the model. Parameters of the model are depicted in Table 1.(color figure online)

Adapted from the models used in Levin and Bull (2004); Styles et al. (2021); Jover et al. (2016), we formulate a mathematical model describing the interaction among two bacterial subpopulations—a phage-susceptible strain and a phage-resistant strain—and a single phage strain using generalized Lotka-Volterra equations (Figure 1).

We denote by *S*(*t*) and *R*(*t*) (CFU/mL), respectively, the population density of the phage-susceptible bacterial strain and the corresponding phage-resistant bacterial strain, and by *P*(*t*) (PFU/mL) the population density of the phage strain. The model is given by the following system of ordinary differential equations:

1 $$\begin{aligned} \begin{aligned} \frac{dS}{dt}&=rS\left( 1-\frac{a_{ss}S + a_{rs}R}{K}\right) - \delta S - \phi PS,\\ \frac{dR}{dt}&= \gamma rR\left( 1-\frac{a_{sr} S+ a_{rr} R}{K}\right) + \delta S - {\epsilon }\phi PR,\\ \frac{dP}{dt}&= \beta \phi (S+{\epsilon }R)P - mP. \end{aligned} \end{aligned}$$

We describe the biological meaning of each term and the assumptions underlying the model structure.

**Bacterial growth and competition.** Each bacterial subpopulation grows logistically. The susceptible strain *S* grows with intrinsic growth rate *r* and carrying capacity *K*. The resistant strain *R* grows at a reduced rate γr, where $\gamma \in (0,1]$ quantifies the fitness cost of resistance. Both subpopulations compete for common resources. In the equation for *S*, the term $a_{ss}S$ captures intraspecific competition among susceptible bacteria, while $a_{rs}R$ captures the competitive effect of resistant bacteria on the susceptible population. Conversely, in the equation for *R*, the term $a_{rr}R$ represents intraspecific competition among resistant bacteria, and $a_{sr}S$ represents the competitive effect of susceptible bacteria on the resistant population. Instead of assuming a single shared interspecific competition coefficient, our model introduces separate coefficients $a_{rs}$ and $a_{sr}$ to reflect the asymmetric nature of interspecific competition. There is no biological reason to expect that the competitive effect of resistant bacteria on susceptible bacteria should equal the reverse; asymmetric competition is the norm rather than the exception in microbial ecology (Tilman 1982).

**Resistance acquisition.** Susceptible bacteria transition to the resistant subpopulation at rate δ, appearing as a loss term -δS in the equation for *S* and a gain term +δS in the equation for *R*. This term aggregates the net effect of spontaneous mutation and any phenotypic switching that produces phage-resistant variants.

**Phage infection and partial resistance.** Phage adsorbs susceptible bacteria at rate ϕ, removing susceptible cells from the population (-ϕPS in equation for *S*). The parameter $\epsilon \in [0,1]$ represents resistance efficiency of resistant bacteria in which phage adsorbs resistant bacteria at a reduced rate $\epsilon \phi$. When $\epsilon \ll 1$, resistant bacteria are rarely infected, corresponding to strong resistance; when ϵ=0, resistance is absolute; and when ϵ=1, there is no resistance. This formulation is motivated by the observation that phage resistance mechanisms—such as modification of surface receptors, restriction-modification systems, or CRISPR-based defense—often reduce but do not completely eliminate phage adsorption (Labrie et al. 2010; Egido et al. 2022). The partial infection of resistant bacteria is represented by the term $-\epsilon \phi PR$ in equation for *R*.

**Phage dynamics.** Following lysis, each infected cell releases β new phage particles (burst size). Both susceptible and resistant bacteria contribute to phage production in proportion to their infection rates, yielding the gain term $\beta \phi (S+\epsilon R)P$ in equation of *P*. Phage particles decay at rate *m*, giving the loss term -mP. Our model assumes instantaneous lysis upon phage adsorption, neglecting the latent period between infection and cell lysis. Given our 48-hour observation window and the specific growth rates of *P. larvae* and *P. aeruginosa*, a continuous model with instantaneous lysis provides a sufficient approximation of the population-level dynamics without the added complexity of delay differential equations (DDEs). The latent period for the phages used in our experiments (typically 20-60 minutes) is substantially shorter than the timescales of resistance evolution observed here (hours to days), and previous modeling studies have shown that instantaneous lysis captures qualitative phage–bacteria dynamics when the focus is on long-term population outcomes rather than short-term oscillatory behavior (Levin and Bull 2004; Payne and Jansen 2001; Weld et al. 2004). Future extensions of this model will incorporate DDEs to examine how latent period duration influences resistance evolution dynamics.

**Carrying capacity.** The carrying capacity *K* is retained as a separate parameter rather than absorbed into the competition coefficients. This preserves the direct biological interpretation of *K* as the maximum sustainable population density, allows *K* to be estimated independently from phage-free control experiments, and maintains balanced parameter magnitudes for numerical stability during model fitting. This parameterization is consistent with the phage–bacteria modeling literature (Levin and Bull 2004; Styles et al. 2021; Jover et al. 2016).

All model parameters and their descriptions are listed in Table 1.

**Table 1:** Parameters and their descriptions for the model system (1)

Parameter	Description	Values [Units]	References
*Bacterial growth parameters*			
*r*	Intrinsic growth rate of *S*	[0.462, 1.65] [$\text {h}^{-1}$]	[20]
γ	Fitness cost of phage resistance	[0, 1] [–]	[4]
*K*	System-wide carrying capacity	$10^9$ [CFU/mL]	[21]
*Competition parameters*			
$a_{ss}$	Intraspecific competition coefficient of *S*	Fitted [–]	–
$a_{rr}$	Intraspecific competition coefficient of *R*	Fitted [–]	–
$a_{rs}$	Interspecific competition coefficient	Fitted [–]	–
	(effect of *R* on *S*)		
$a_{sr}$	Interspecific competition coefficient	Fitted [–]	–
	(effect of *S* on *R*)		
*Resistance acquisition*			
δ	Rate of resistance acquisition	$[10^{-6}, 10^{-3}]$ [$\text {h}^{-1}$]	[4]
*Phage parameters*			
ϕ	Adsorption rate of phage *P* to *S*	$[10^{-9}, 10^{-6}]$ [mL· $\text {PFU}^{-1}\cdot$ $\text {h}^{-1}$]	[1, 4, 19]
ϵ	Resistance efficiency	$[10^{-5},0.5]$ [-]	This study
β	Burst size of phage *P*	[10, 100] [PFU· $\text {CFU}^{-1}$]	[1, 4]
*m*	Decay rate of phage *P*	$[1.5 \times 10^{-3}, 0.1]$ [$\text {h}^{-1}$]	[22]
*Initial conditions*			
$S_0$	Initial density of *S*	Fitted [CFU/mL]	–
$R_0$	Initial density of *R*	Fitted [CFU/mL]	–
$P_0$	Initial density of *P*	Experiment [PFU/mL]	–

### Experimental data used for model fitting

**Fig. 2:**
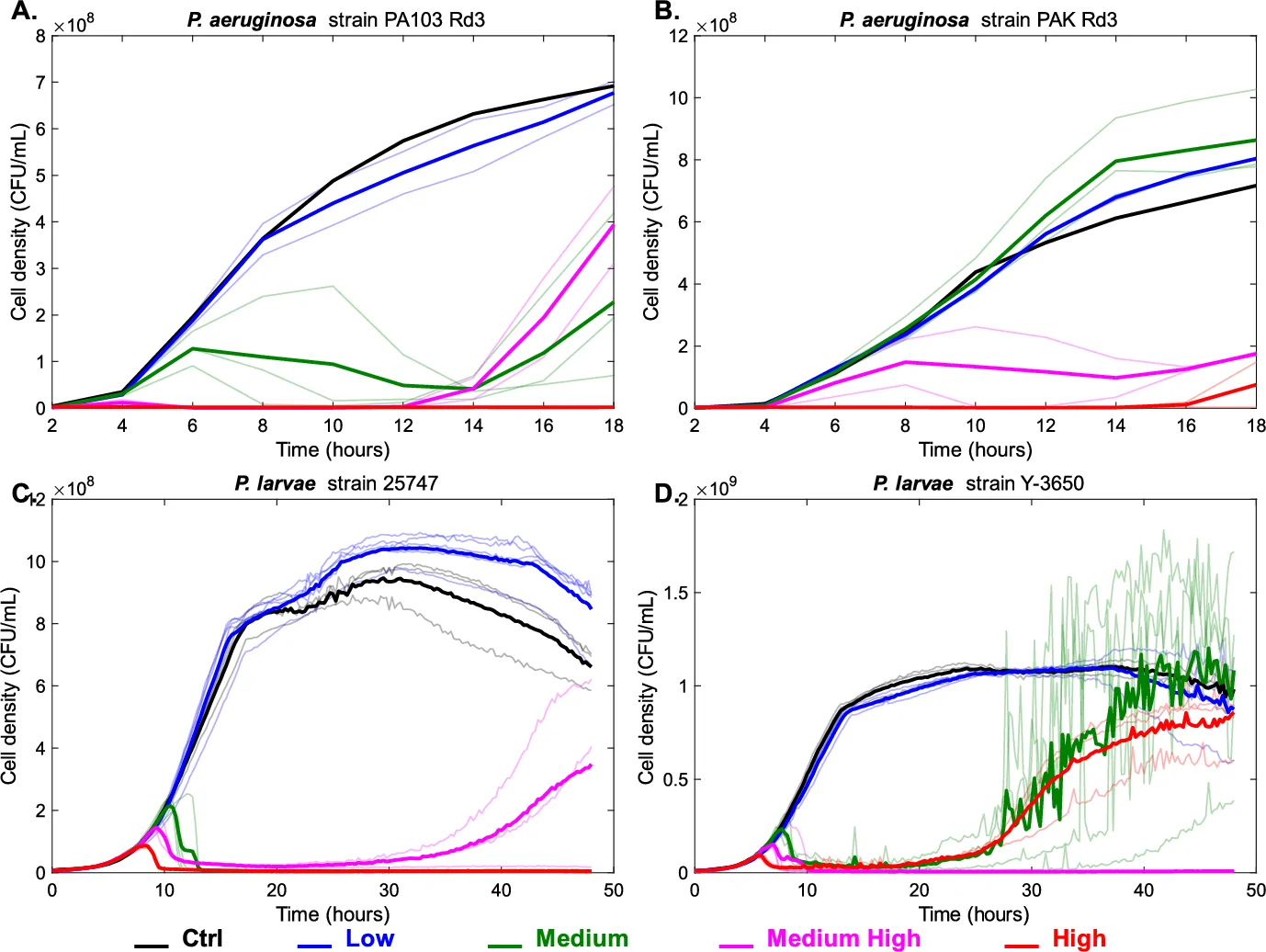
Dynamics of growth experimental data of four host strains that exhibit two-phase growth patterns. Panels A and B, respectively, show growth dynamics of *P. aeruginosa* PA103 and PAK at round 3 of Appelmans protocol with different dilutions of a cocktail of phages. Panels C and D, respectively, represent growth dynamics of *P. larvae* 25747 and *P. larvae* Y-3650 with different dilutions of phage Fern_IDv1 (3 replicates for each dilution).(color figure online)

To measure how the multiplicity of infection (MOI) impacts resistance evolution, a series of growth experiments were performed on a shaken, incubating microplate reader. We chose eight different hosts (5 strains of *P. aeruginosa* and 3 strains of *P. larvae*) with four different phage challenges (Table 2). These combinations were chosen because these challenges always resulted in phage-resistant colonies overnight when tested on solid agar plates (Spencer et al. 2025; Peters et al. 2024). Unexpectedly, for most (8/14) challenges, we did not observe resistance evolution in liquid culture at any dilution of phage (Table 2). We considered resistance evolution to have occurred when bacterial growth was delayed compared to control or when two periods of bacterial growth were measured (Figure 2). These two phases of growth included an immediate initial growth period that matched the no-phage control that was followed by bacterial death (drop in cell density) and then a rebound in bacterial growth (presumably the rise of a resistant variant(s), Figure 2). The phage-host combinations where we observed these dynamics are labeled “two-phase growth” in (Table 2). The *P. aeruginosa* experiments were initially designed to expand the host range of a phage cocktail through experimental evolution (Appelmans protocol). Thus, *P. aeruginosa* strains were challenged with a mix of three phages. We observed two-phase growth dynamics in strains PA103 and PAK. Strains PA14 and PA01 also grew after a delay, indicating that resistant strains evolved. For *P. larvae*, we did not observe any strains with only delayed growth, only two-phase growth. Therefore, we chose to use only *P. aeruginosa* strains with this two-phase growth in our modeling. The *P. larvae* experiments were performed with one phage strain at a time. Infections of *P. larvae* did not always result in two-phase growth, but instead varied by MOI. For *P. aeruginosa*, we observed two-phase growth in only the medium and medium-high MOIs for PA103, and only the medium-high and high MOIs for PAK. For *P. larvae*, we observed two-phase growth in only the medium-level MOI for strain 25747 and in some low MOI replicates, all medium MOI replicates, and all high MOI replicates for strain 3650. Notably, suppression was non-monotonic in MOI for the *P. larvae* strains—an intermediate phage dose produced stronger suppression than a higher dose—an unexpected pattern that our model reproduces only with treatment-specific competition coefficients and whose biological basis we examine further in the Discussion and Experimental limitations.

**Table 2:** Results of growth experiments using eight hosts with four different phages

Strains	Fern_IDv1	Scot_IDv1	Xeni_IDv1	Cocktail
*P. larvae* Y-3650	two-phase growth	Complete suppression	Complete suppression	Not tested
*P. larvae* 25747	two-phase growth	Complete suppression	Complete suppression	Not tested
*P. larvae* B-2605	Complete suppression	Complete suppression	Complete suppression	Not tested
*P. aeruginosa* PA103	Not tested	Not tested	Not tested	two-phase growth
*P. aeruginosa* PA14	Not tested	Not tested	Not tested	Delayed growth
*P. aeruginosa* PA01	Not tested	Not tested	Not tested	Delayed growth
*P. aeruginosa* PAK	Not tested	Not tested	Not tested	two-phase growth
*P. aeruginosa* PDO300	Not tested	Not tested	Not tested	Complete suppression

Among the results of growth experiments using 8 different hosts (5 strains of *Pseudomonas aeruginosa* and 3 strains of *Paenibacillus larvae*) with four different phages (Fern_IDv1, Scot_IDv1, Xeni_IDv1, and cocktail), we observed two-phase growth dynamics (an immediate growth period followed by bacterial death and then a rebound in bacterial growth) as well as delayed growth dynamics (indicating resistant strains evolved) in strains PA103, PAK, Y-3650, and 25747. We therefore chose to use these four strains in fitting our mathematical model. Our goal was to measure how the multiplicity of infection (MOI) impacts resistance evolution.

To examine resistance evolution in the chosen four strains using our mathematical model, we classified their experimental growth data into 5 groups (Ctrl, Low, Medium, Medium High, High) based on the initial MOI and their growth patterns (see Figure 2).

A. PA103 Rd3 challenged with phage cocktail: Control group included phage-free bacterial cultures; Low group averaged $10^{-9}$ and $10^{-8}$ dilutions that showed minimal phage impact; Medium group combined $10^{-7}$, $10^{-6}$, and $10^{-5}$ dilutions exhibiting two-phase growth patterns; Medium High group averaged $10^{-4}$ and $10^{-3}$ dilutions showing delayed growth; and High group encompassed $10^{-2}$, $10^{-1}$, and undiluted phage stocks resulting in complete suppression.
B. PAK Rd3 with phage cocktail followed a different grouping strategy: Low included three dilutions ($10^{-9}$, $10^{-8}$, $10^{-7}$) that maintained growth similar to controls; Medium averaged $10^{-6}$, $10^{-5}$, and $10^{-4}$ dilutions producing two-phase growth; Medium High combined $10^{-3}$ and $10^{-2}$ dilutions showing delayed growth; while High group included only $10^{-1}$ and undiluted treatments causing complete suppression.
C. 25747 challenged with Fern IDv1 phage: Control averaged three phage-free replicates; Low group uniquely combined five replicates from $10^{-9}$ (all three replicates) and $10^{-8}$ (two replicates) dilutions; Medium included one replicate from $10^{-8}$ and all three from $10^{-7}$ dilutions; Medium High averaged all three $10^{-6}$ replicates; and High encompassed all three $10^{-5}$ replicates.
D. Y-3650 with Fern IDv1 showed the most distinct grouping: Low included only the three $10^{-9}$ replicates; Medium combined one $10^{-8}$ replicate with all three $10^{-7}$ replicates; Medium High averaged one $10^{-8}$ replicate with all three $10^{-6}$ replicates; and High included all three $10^{-5}$ replicates.

These strain-specific grouping strategies reflect the different sensitivities of bacterial species to phage pressure and enabled identification of MOI-dependent competitive dynamics driving resistance evolution.

### Parameter estimation and model fitting method

#### Two-stage global fitting procedure

Parameter estimation was performed in two stages. In the first stage, we estimated bacterial growth parameters from phage-free control data. In the second stage, we fixed these growth parameters and simultaneously fitted the full model to the four phage treatment groups (Low, Medium, Medium High, and High) using a global fitting approach.

**Stage 1: Control fitting.** For each bacterial strain, we fitted the logistic growth equation

$$\begin{aligned} \frac{dS}{dt}=rS\left( 1-\frac{a_{ss}S}{K}\right) \end{aligned}$$

to the mean control (phage-free) growth data by nonlinear least squares, estimating three parameters: the intrinsic growth rate *r*, the intraspecific competition coefficient $a_{ss}$, and the initial susceptible density $S_0$. The carrying capacity was fixed at $K=10^9$ CFU/mL based on independent estimates from stationary-phase densities across all strains (Levin and Bull 2004; Styles et al. 2021; Jover et al. 2016). For *P. aeruginosa* strains (PA103 Rd3 and PAK Rd3), where growth data were recorded in CFU/mL over 9 time points spanning 2–18 hours, $S_0$ was back-extrapolated to t=0 from the earliest available measurement. For *P. larvae* strains (Y-3650 and 25747), optical density measurements ($\text {OD}_{600}$) were converted to CFU/mL using the calibration factor 1 $\text {OD}_{600}\approx 10^9$ CFU/mL, yielding 193 time points over 48 hours per treatment group.

**Stage 2: Global fitting to phage treatment data.** With *r*, $a_{ss}$, $S_0$ and *K* fixed at their control-estimated values, we simultaneously fitted the model to the grouped mean growth data of all four treatments. The term “global fitting”refers to the simultaneous optimization of a single parameter vector across all four treatment datasets, minimizing a combined objective function (described below). This approach ensures that shared parameters are constrained by all treatments simultaneously, while treatment-specific parameters capture MOI-dependent variation.

#### Parameter classification: shared and treatment-specific

A central modeling decision is which parameters should be shared across MOI treatments and which should be allowed to vary. We classify the parameters into three tiers based on their biological roles.

**Parameters fixed from control data.** The intrinsic growth rate *r*, the intraspecific competition coefficient of susceptible bacteria $a_{ss}$, the initial susceptible density $S_0$, and the carrying capacity *K* describe the growth of the susceptible strain in the absence of phage. These were estimated from phage-free control experiments and held fixed across all treatments.

**Parameters shared across treatments.** The intraspecific competition coefficient of resistant bacteria $a_{rr}$, the burst size β, and the phage decay rate *m* describe intrinsic properties of the resistant subpopulation and of the phage that should not depend on the initial phage dose. These three parameters were constrained to take a single value across all four treatments within each strain.

**Parameters allowed to vary across treatments.** The remaining parameters—interspecific competition coefficients $a_{rs}$ and $a_{sr}$, resistance efficiency ϵ, fitness cost γ, resistance acquisition rate δ, adsorption rate ϕ, and the initial resistant density $R_0$—were allowed to take treatment-specific values. The biological rationale for permitting these parameters to vary, together with its interpretation in terms of coevolutionary arms-race dynamics and phage-mediated indirect interactions, is developed in the Discussion (Section 4); here we restrict attention to defining the two parameterization schemes that are compared.

#### Two model versions

Based on these considerations, we compared two parameterization schemes:

- **M1 (5 shared parameters):** The shared parameters are $a_{rs}$, $a_{sr}$, $a_{rr}$, β, and *m*. The treatment-specific parameters are ϵ, γ, δ, ϕ, and $R_0$, yielding 5+5×4=25 free parameters.
- **M2 (3 shared parameters):** The shared parameters are $a_{rr}$, β, and *m*. The treatment-specific parameters are $a_{rs}$, $a_{sr}$, ϵ, γ, δ, ϕ, and $R_0$, yielding 3+7×4=31 free parameters.

For PA103 Rd3 and PAK Rd3, M1 produced adequate fits, so only M1 results are reported for these strains (Table 3). For Y-3650 and 25747, M1 produced poor fits for specific treatments (Tables 4 and 5), motivating the M2 parameterization. Both M1 and M2 are fitted for Y-3650 and 25747, and formal model comparison was performed (Section 3.1).

**Table 3:** Fitted parameter values and goodness-of-fit statistics for *P. aeruginosa* strains PA103 Rd3 and PAK Rd3, each challenged with a phage cocktail. Parameters are organized into three tiers: (i) parameters fixed from phage-free control data, (ii) parameters shared across all four MOI treatments, and (iii) treatment-specific parameters. Both strains are reported under model M1, in which the five parameters $a_{rs}$, $a_{sr}$, $a_{rr}$, β, and *m* are shared across treatments. The initial phage densities $P_0$ for the Low, Medium, Med-High, and High treatments are 0.10, 1.0, 10, and 100 PFU/mL for both strains.

		**PA103 Rd3**				**PAK Rd3**			
	Parameter	Low	Med	Med-High	High	Low	Med	Med-High	High
*Fixed parameters*									
	*r*	1.2217				1.2801			
	$a_{ss}$	1.6143				1.6035			
	$S_0$	$6.00 \times 10^{5}$				$1.03 \times 10^{5}$			
	*K*	$1.00 \times 10^{9}$				$1.00 \times 10^{9}$			
**Model M1** (5 shared parameters)									
*Shared parameters*									
	$a_{rs}$	0.4359				2.043			
	$a_{sr}$	0.6354				1.640			
	$a_{rr}$	1.467				1.218			
	β	15.52				15.76			
	*m*	0.4624				0.5000			
*Treatment-specific parameters*									
	ϵ	0.00969	0.0517	0.00629	0.00319	0.00580	$1.00{\times }10^{-5}$	0.0440	0.0317
	γ	0.665	0.431	0.510	$2.72{\times }10^{-4}$	0.512	0.529	0.568	0.372
	δ	$3.92{\times }10^{-5}$	$3.87{\times }10^{-4}$	$1.00{\times }10^{-6}$	$5.61{\times }10^{-4}$	$1.00{\times }10^{-3}$	$1.00{\times }10^{-3}$	$1.00{\times }10^{-3}$	$1.00{\times }10^{-6}$
	ϕ	$4.49{\times }10^{-9}$	$4.13{\times }10^{-9}$	$6.26{\times }10^{-8}$	$2.37{\times }10^{-7}$	$5.30{\times }10^{-9}$	$5.45{\times }10^{-9}$	$5.33{\times }10^{-9}$	$3.19{\times }10^{-8}$
	$R_0$	$5.68{\times }10^{6}$	$6.09{\times }10^{6}$	$7.79{\times }10^{4}$	$2.22{\times }10^{6}$	$5.03{\times }10^{6}$	$5.03{\times }10^{6}$	$1.77{\times }10^{6}$	$2.06{\times }10^{5}$
*Goodness of fit*									
	$R^2$	0.987	0.881	0.974	0.816	0.995	0.994	0.760	0.818
	RMSE	$2.73{\times }10^{7}$	$2.22{\times }10^{7}$	$2.07{\times }10^{7}$	$1.12{\times }10^{5}$	$2.10{\times }10^{7}$	$2.55{\times }10^{7}$	$2.79{\times }10^{7}$	$9.74{\times }10^{6}$
	Mean $R^2$	0.914				0.892			
	AIC	1262				1268			
	BIC	1301				1307			

Model M1 produced adequate fits for both strains (mean $R^2 = 0.914$ for PA103 Rd3; mean $R^2 = 0.892$ for PAK Rd3), and the treatment-specific parameterization M2 was therefore not pursued

**Table 4:** Fitted parameter values and goodness-of-fit statistics for *P. larvae* strain Y-3650 challenged with phage Fern IDv1. Parameters are organized into three tiers: (i) parameters fixed from control data, (ii) parameters shared across all four MOI treatments, and (iii) treatment-specific parameters. Model M1 constrains five parameters as shared ($a_{rs}$, $a_{sr}$, $a_{rr}$, β, *m*), while model M2 constrains only three ($a_{rr}$, β, *m*) and allows the interspecific competition coefficients $a_{rs}$ and $a_{sr}$ to vary across treatments. The initial phage densities $P_0$ for the Low, Medium, Med-High, and High treatments are 0.347, 3.47, 34.7, and 347 PFU/mL, respectively. Information-theoretic model comparison yields $\Delta \text {AIC} = \text {AIC}_{M2} - \text {AIC}_{M1} = -753$ and $\Delta \text {BIC} = -725$, providing decisive support for the treatment-specific parameterization M2.

	Parameter	Low	Medium	Med-High	High
*Fixed parameters*					
	*r*	0.4699			
	$a_{ss}$	0.9411			
	$S_0$	$9.00 \times 10^{6}$			
	*K*	$1.00 \times 10^{9}$			
**M1** (5 shared parameters)					
*Shared parameters*					
	$a_{rs}$	11.62			
	$a_{sr}$	0.1001			
	$a_{rr}$	0.9231			
	β	18.77			
	*m*	0.4886			
*Treatment-specific parameters*					
	ϵ	0.0324	0.00660	0.00500	$1.00 \times 10^{-4}$
	γ	1.000	0.611	0.400	0.418
	δ	$1.00 \times 10^{-3}$	$5.76 \times 10^{-4}$	$1.00 \times 10^{-4}$	$1.00 \times 10^{-3}$
	ϕ	$1.00 \times 10^{-9}$	$1.80 \times 10^{-9}$	$1.00 \times 10^{-8}$	$4.99 \times 10^{-7}$
	$R_0$	$1.00 \times 10^{6}$	$2.23 \times 10^{4}$	$1.00 \times 10^{5}$	$1.00 \times 10^{6}$
*Goodness of fit*					
	$R^2$	0.858	0.972	-19.71	0.947
	RMSE	$1.40 \times 10^{8}$	$6.81 \times 10^{7}$	$1.30 \times 10^{8}$	$7.26 \times 10^{7}$
	Mean $R^2$	-4.233			
	AIC	28607			
	BIC	28723			
**M2** (3 shared parameters)					
*Shared parameters*					
	$a_{rr}$	0.9248			
	β	18.75			
	*m*	0.4801			
*Treatment-specific parameters*					
	$a_{rs}$	4.484	164.3	271.1	9.318
	$a_{sr}$	0.03367	0.05642	232.8	6.451
	ϵ	0.0369	0.0145	0.129	0.0112
	γ	0.999	0.619	0.769	0.346
	δ	$1.00 \times 10^{-3}$	$6.57 \times 10^{-4}$	$1.34 \times 10^{-5}$	$2.44 \times 10^{-4}$
	ϕ	$1.00 \times 10^{-9}$	$1.75 \times 10^{-9}$	$2.71 \times 10^{-9}$	$5.99 \times 10^{-9}$
	$R_0$	$3.47 \times 10^{6}$	$1.86 \times 10^{4}$	$1.46 \times 10^{6}$	$6.13 \times 10^{6}$
*Goodness of fit*					
	$R^2$	0.934	0.973	0.712	0.966
	RMSE	$9.55 \times 10^{7}$	$6.72 \times 10^{7}$	$1.53 \times 10^{7}$	$5.80 \times 10^{7}$
	Mean $R^2$	0.896			
	AIC	27854			
	BIC	27998			

#### Objective function and error model.

The departure between model predictions and experimental data was quantified by the sum of squared residuals. For the global fit across T=4 treatments, the objective function is

$$\begin{aligned} L(\theta ):=\sum _{j=1}^T\sum _{i=1}^{n_j}[y_j(t_i)-\hat{y}_j(t_i;\theta )]^2 \end{aligned}$$

where $y_j(t_i)$ is the observed mean total cell density (in CFU/mL) at time point $t_i$ in treatment *j*, $\hat{y}_j(t_i;\theta )=S_j(t_i;\theta )+R_j(t_i;\theta )$ is the model-predicted total cell density obtained by numerically solving the ODE system, $n_j$ is the number of time points in treatment *j*, and θ is the full parameter vector containing both shared and treatment-specific components. The residuals were not weighted, which assumes approximately homogeneous variance across treatments and time points. This choice is appropriate because all treatments use the same measurement method and operate in the same density range, and because the $\text {OD}_{600}$-to-CFU conversion applies a uniform scaling factor across all data.

#### Optimization algorithm and numerical implementation

All fitting was performed in MATLAB (R2023b, MathWorks). Parameter estimation was carried out using MATLAB’s lsqnonlin function, which implements a trust-region-reflective algorithm for bound-constrained nonlinear least squares. This algorithm iteratively adjusts the parameter vector to minimize the sum of squared residuals, using an approximation to the Jacobian matrix to determine search directions and step sizes within a trust region.

Upper and lower bounds were imposed on all parameters to enforce biological plausibility (see Table 1 for parameter ranges). The ODE system was integrated at each function evaluation using MATLAB’s ode15s solver, a variable-order, variable-step-size method based on numerical differentiation formulas, appropriate for the stiff dynamics present in the phage-bacteria system. Integration tolerances were set to $\text {RelTol}=10^{-8}$ and $\text {AbsTol}=10^{-10}$, with the nonnegativity constraint enforced on all three state variables (*S*, *R*, *P*). The lsqnonlin optimizer was configured with a maximum of 10,000 function evaluations and 5,000 iterations, with function tolerance and step tolerance both set to $10^{-12}$.

#### Goodness of fit and model comparison

The quality of each fit was assessed using the coefficient of determination $R^2$, computed both per treatment and overall:

$$\begin{aligned} R_j^2=1-\frac{SS_{res,j}}{SS_{tot,j}} \end{aligned}$$

where $SS_{res,j}=\sum _{i=1}^{n_j}[y_j(t_i)-\hat{y}_j(t_i)]^2$ is the residual sum of squares and $SS_{tot,j}=\sum _{i=1}^{n_j}[y_j(t_i)-\bar{y}_j]^2$ is the total sums of squares for treatment *j* (with $\bar{y}_j=\sum _{i=1}^{n_j}y_j(t_i)/n_j$). The overall $R^2$ was computed analogously using the pooled residuals and pooled total variation across all treatments. The root mean squared error $\text {RMSE}=\sqrt{SS_{res}/n}$ was also reported for each treatment, where $SS_{res}=\sum _{j=1}^T SS_{res,j}$ and *n* is the number of data points.

For the two strains where both M1 and M2 were fitted (Y-3650 and 25747), formal model comparison was performed using the Akaike Information Criterion (AIC) and Bayesian Information Criterion (BIC):

$$\begin{aligned} \text {AIC}=n\cdot \ln (SS_{res}/n) + 2k,\qquad \text {BIC}=n\cdot \ln (SS_{res}/n)+k\cdot \ln (n), \end{aligned}$$

where *n* is the total number of data points across all four treatments and *k* is the number of free parameters. The difference $\Delta \text {AIC}=\text {AIC}_{\text {M2}}-\text {AIC}_{\text {M1}}$ (and analogously for BIC) quantifies the relative evidence: values more negative than -10 are conventionally interpreted as decisive evidence in favor of M2 (Burnham and Anderson 2002).

To further assess whether treatment-specific interspecific competition parameters are required, we performed a profile likelihood analysis for $a_{rs}$ and $a_{sr}$ in Y-3650 and 25747 (Section 3.2).

## Results

### Model fitting results

We fitted model  (1) to experimental growth data from four bacterial strains that exhibited resistance-associated dynamics across phage treatments: *P. aeruginosa* PA103 Rd3 and PAK Rd3 challenged with the phage cocktail, and *P. larvae* Y-3650 and 25747 challenged with Fern IDv1 (Figure 2). For each strain, bacterial growth parameters were first estimated from phage-free control data (Stage 1), then held fixed during the global fit to the four phage-treatment groups (Stage 2). Tables 3–5 report the full set of fitted parameter values and goodness-of-fit statistics for all four strains.

**Table 5:** Fitted parameter values and goodness-of-fit statistics for *P. larvae* strain 25747 challenged with phage Fern IDv1. The table is organized identically to Table 4. Model M1 constrains five parameters as shared ($a_{rs}$, $a_{sr}$, $a_{rr}$, β, *m*), while model M2 constrains only three ($a_{rr}$, β, *m*) and allows $a_{rs}$ and $a_{sr}$ to vary across treatments. The initial phage densities $P_0$ for the Low, Medium, Med-High, and High treatments are 0.203, 2.03, 20.3, and 203 PFU/mL, respectively. Under M1, the Medium and High treatments are poorly fitted ($R^2 = 0.438$ and $R^2 = -0.122$, respectively), and the M1 shared value of $a_{sr} = 2.341$ is approximately 1000-fold larger than the treatment-specific optima identified by profile likelihood analysis (Section 3.2), a signature of compensatory parameter inflation in over-constrained models. Information-theoretic model comparison yields $\Delta \text {AIC} = -458$ and $\Delta \text {BIC} = -430$, decisively favoring the treatment-specific parameterization M2.

	Parameter	Low	Medium	Med-High	High
*Fixed parameters*					
	*r*	0.3730			
	$a_{ss}$	1.0963			
	$S_0$	$7.00 \times 10^{6}$			
	*K*	$1.00 \times 10^{9}$			
**M1** (5 shared parameters)					
*Shared parameters*					
	$a_{rs}$	66.46			
	$a_{sr}$	2.341			
	$a_{rr}$	1.003			
	β	38.33			
	*m*	0.2738			
*Treatment-specific parameters*					
	ϵ	0.0276	0.0432	0.0524	0.0680
	γ	0.976	0.999	0.402	0.952
	δ	$9.98 \times 10^{-4}$	$1.00 \times 10^{-3}$	$9.99 \times 10^{-4}$	$1.00 \times 10^{-3}$
	ϕ	$1.00 \times 10^{-9}$	$7.47 \times 10^{-9}$	$1.09 \times 10^{-9}$	$1.23 \times 10^{-8}$
	$R_0$	$7.20 \times 10^{6}$	$4.59 \times 10^{6}$	$1.40 \times 10^{6}$	$6.00 \times 10^{6}$
*Goodness of fit*					
	$R^2$	0.992	0.438	0.973	-0.122
	RMSE	$3.40 \times 10^{7}$	$3.27 \times 10^{7}$	$1.41 \times 10^{7}$	$1.84 \times 10^{7}$
	Mean $R^2$	0.571			
	AIC	26429			
	BIC	26545			
**M2** (3 shared parameters)					
*Shared parameters*					
	$a_{rr}$	1.003			
	β	38.34			
	*m*	0.2733			
*Treatment-specific parameters*					
	$a_{rs}$	66.29	201.3	33.09	223.1
	$a_{sr}$	2.342	1.195	0.02524	1.775
	ϵ	0.0276	0.0433	0.0525	0.0682
	γ	0.974	1.000	0.402	0.955
	δ	$9.99 \times 10^{-4}$	$1.00 \times 10^{-3}$	$1.00 \times 10^{-3}$	$1.00 \times 10^{-3}$
	ϕ	$1.00 \times 10^{-9}$	$1.26 \times 10^{-8}$	$1.01 \times 10^{-9}$	$1.29 \times 10^{-8}$
	$R_0$	$7.27 \times 10^{6}$	$4.57 \times 10^{6}$	$1.40 \times 10^{6}$	$6.02 \times 10^{6}$
*Goodness of fit*					
	$R^2$	0.992	0.915	0.979	0.912
	RMSE	$3.41 \times 10^{7}$	$1.27 \times 10^{7}$	$1.25 \times 10^{7}$	$5.15 \times 10^{6}$
	Mean $R^2$	0.950			
	AIC	25971			
	BIC	26115			

**Fig. 3:**
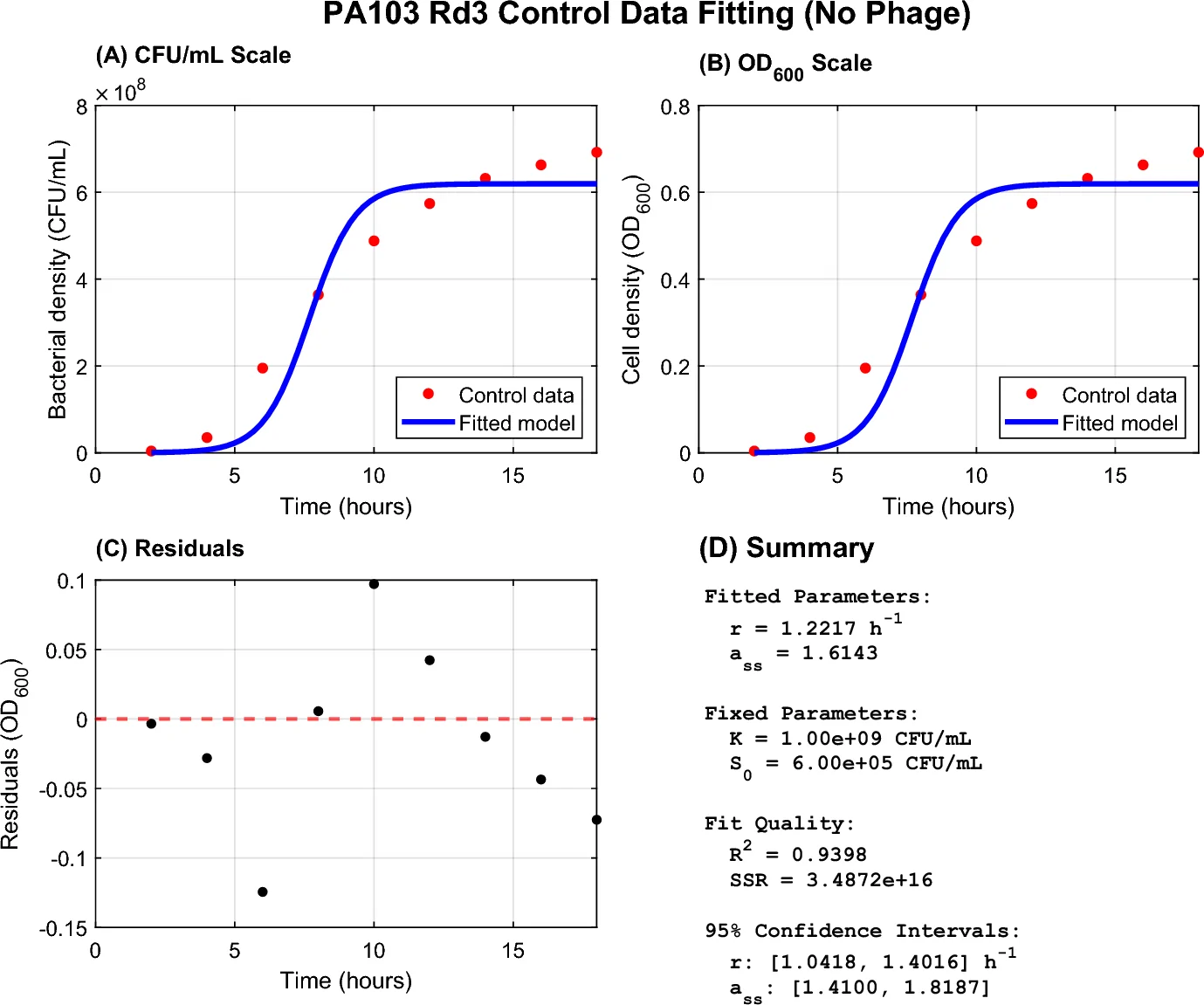
Control fit for *P. aeruginosa* strain PA103 Rd3 in the absence of phage. Panel (A) shows the bacterial growth data on the CFU/mL scale with the fitted logistic curve. Panel (B) shows the corresponding fit on the $\text {OD}_{600}$ scale. Panel (C) presents the residuals, and Panel (D) reports the estimated control parameters and goodness-of-fit statistics.(color figure online)

**Control fitting.** Logistic growth curves fitted the phage-free control data well across four strains (Figures 3, 5, 7, and 9). The two *P. aeruginosa* strains exhibited intrinsic growth rates approximately three times higher than those of the *P. larvae* strains (PA103 Rd3: r=1.22 $\text {h}^{-1}$; PAK Rd3: r=1.28 $\text {h}^{-1}$; Y-3650: r=0.47 $\text {h}^{-1}$; 25747: r=0.37 $\text {h}^{-1}$), consistent with the known growth characteristics of these genera. The intraspecific competition coefficients $a_{ss}$ ranged from 0.94 to 1.61, with *P. aeruginosa* strains showing values above unity ($a_{ss}\approx 1.6$) and *P. larvae* strains showing values near unity ($a_{ss}\approx 0.9$–1.1). Control fits achieved $R^2\ge 0.94$ for all strains.

**Fig. 4:**
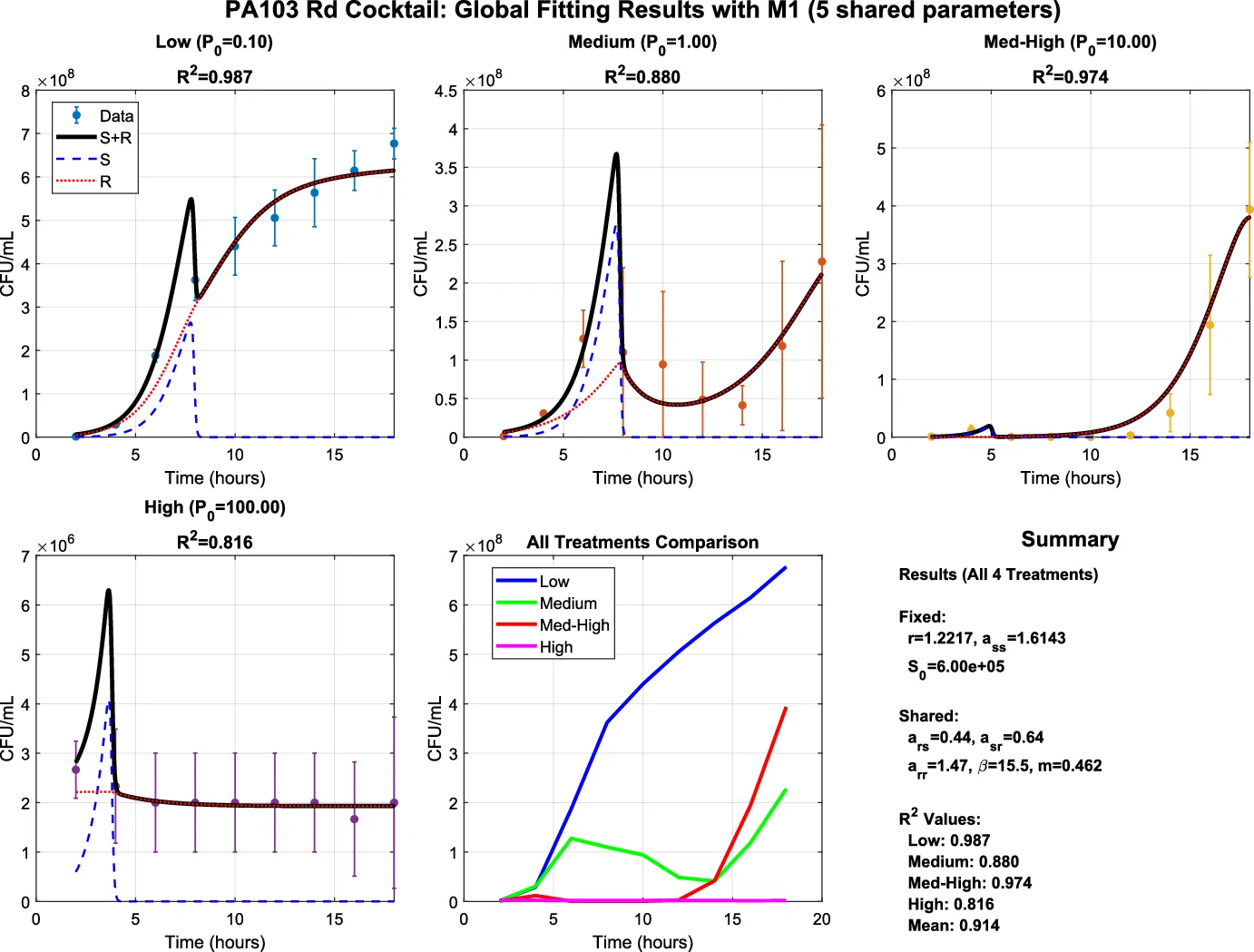
Global fit of M1 model to the four phage treatment groups for *P. aeruginosa* strain PA103 Rd3. Panels show the fitted dynamics for the Low, Medium, Med-High, and High treatments. The comparison panel summarizes the treatment-specific trajectories, and the summary panel reports the shared parameter estimates and goodness-of-fit statistics.(color figure online)

**Global fitting:** ***P. aeruginosa*** **strains (M1).** For PA103 Rd3 and PAK Rd3, model M1—in which $a_{rs}$, $a_{sr}$, $a_{rr}$, β, and *m* are shared across all four treatments—produced adequate fits (Figures 4 and 6; Table 3). PA103 Rd3 achieved a mean treatment $R^2$ of 0.914, with per-treatment values ranging from 0.816 (High) to 0.987 (Low). The model captured the characteristic two-phase pattern in the Medium treatment—an initial rise driven by susceptible bacteria, a crash following phage amplification, and a subsequent rebound of resistant bacteria—as well as the delayed growth and suppression dynamics in the Med-High and High treatments, respectively. PAK Rd3 showed a comparable mean $R^2$ of 0.892, ranging from 0.760 (Med-High) to 0.995 (Low). In both strains, the shared interspecific competition coefficients under M1 adequately captured the observed dose-response patterns without requiring treatment-specific values. The fitted shared competition coefficients differed between strains: PA103 Rd3 yielded $a_{rs}=0.44$ and $a_{sr}=0.64$, indicating a competitive environment in which neither subpopulation strongly dominates the other, while PAK Rd3 yielded $a_{rs}=2.04$ and $a_{sr}=1.64$, indicating stronger mutual competitive effects.

**Fig. 5:**
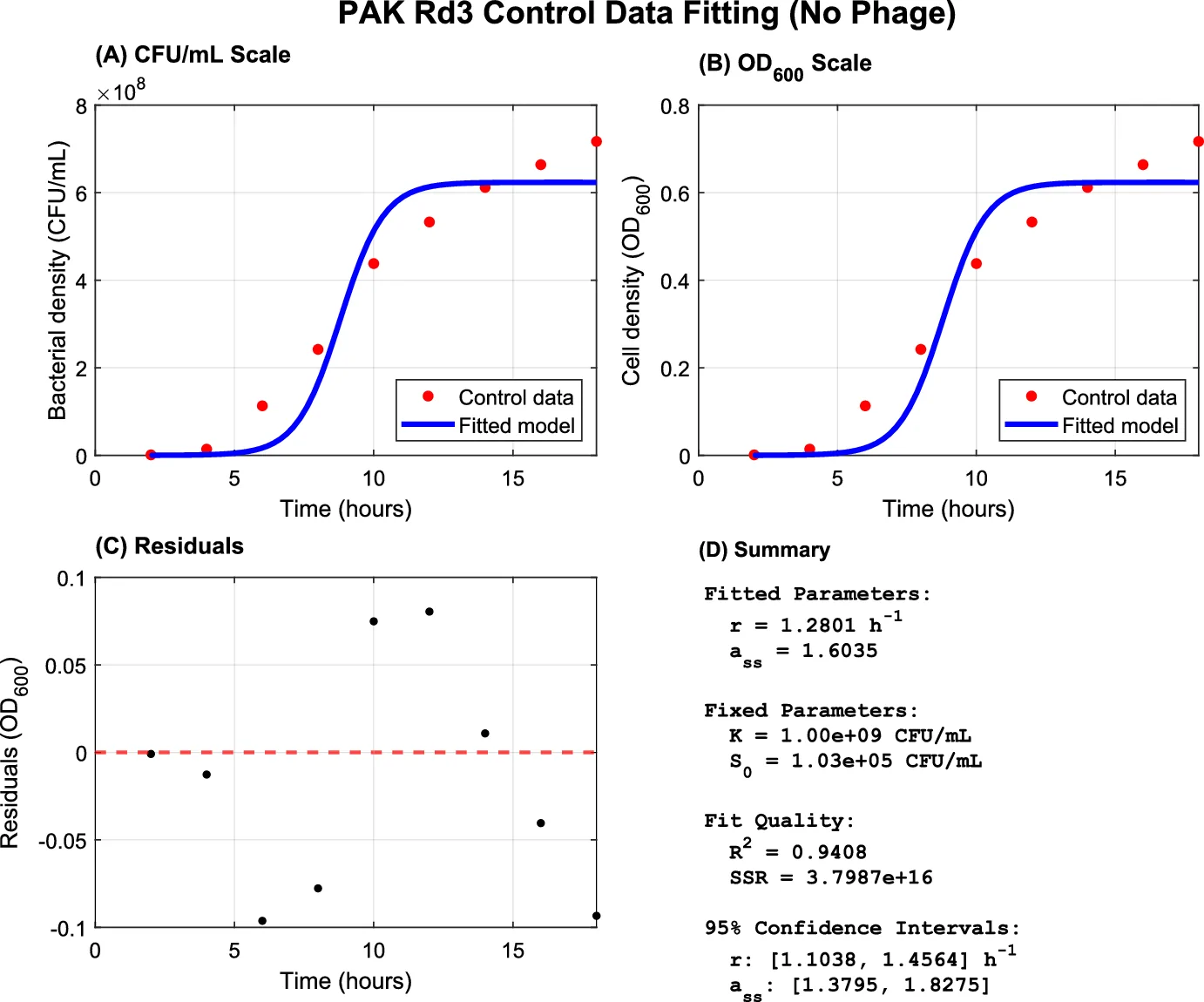
Control fit for *P. aeruginosa* strain PAK Rd3 in the absence of phage. Panel (A) shows the bacterial growth data on the CFU/mL scale with the fitted logistic curve. Panel (B) shows the corresponding fit on the $\text {OD}_{600}$ scale. Panel (C) presents the residuals, and Panel (D) reports the estimated control parameters and goodness-of-fit statistics.(color figure online)

**Fig. 6:**
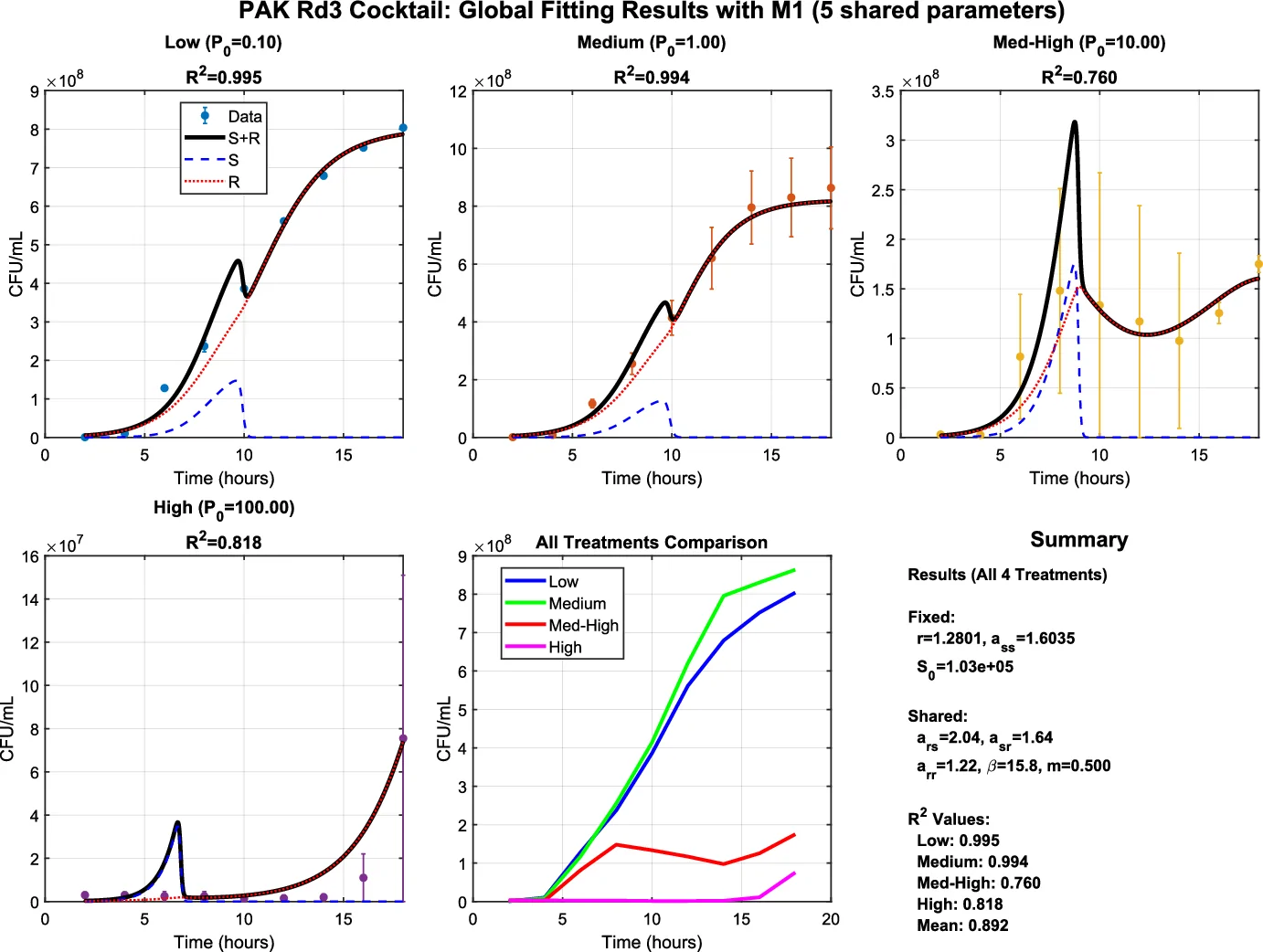
Global fit of model (1) to the four phage treatment groups for *P. aeruginosa* strain PAK Rd3. Panels show the fitted dynamics for the Low, Medium, Med-High, and High treatments. The comparison panel summarizes the treatment-specific trajectories, and the summary panel reports the shared parameter estimates and goodness-of-fit statistics.(color figure online)

**Fig. 7:**
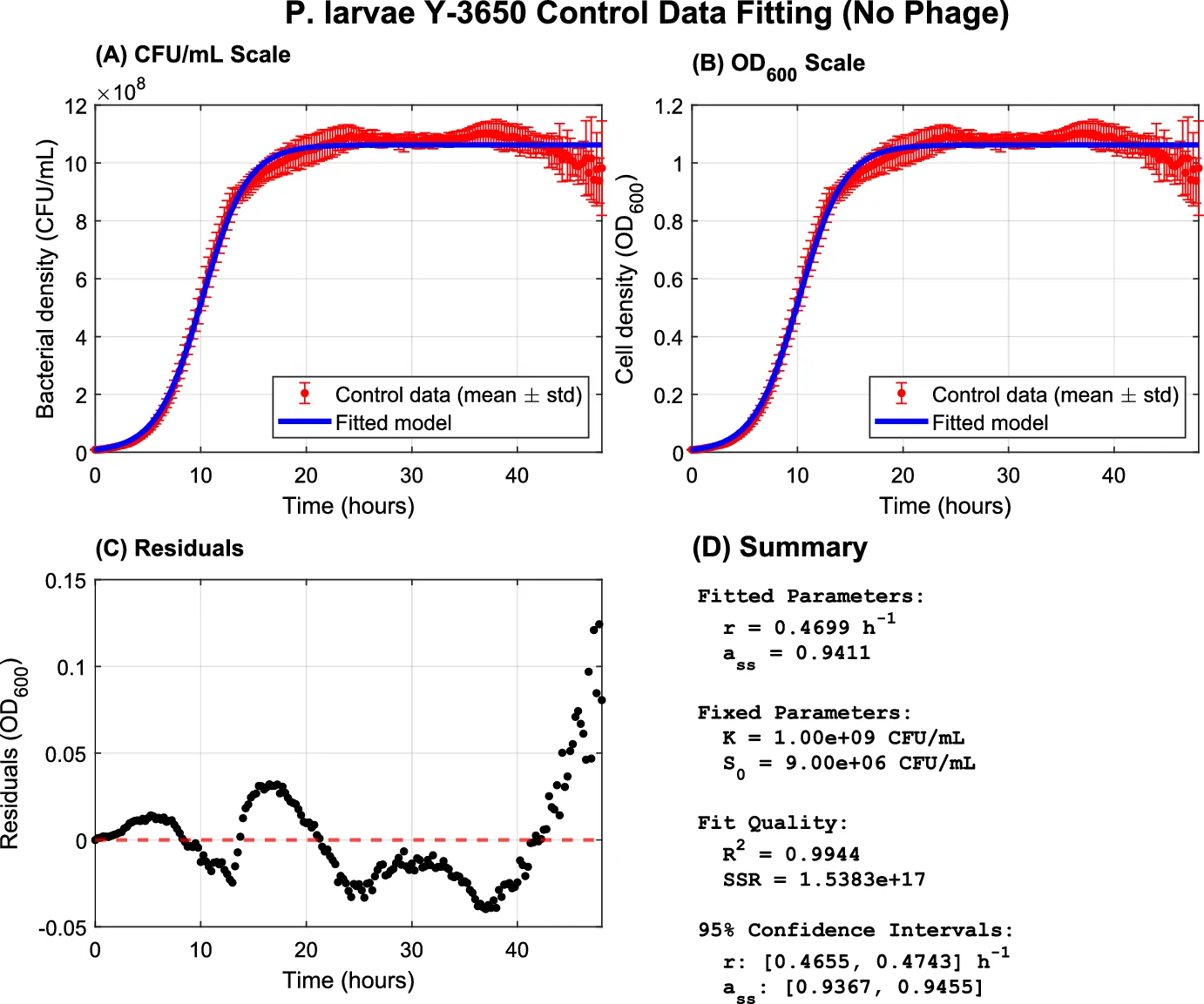
Control fit for *P. larvae* strain Y-3650 in the absence of phage. Panel (A) shows the bacterial growth data on the CFU/mL scale with the fitted logistic curve. Panel (B) shows the corresponding fit on the $\text {OD}_{600}$ scale. Panel (C) presents the residuals, and Panel (D) reports the estimated control parameters and goodness-of-fit statistics.(color figure online)

**Global fitting:** ***P. larvae*** **strains (M1 vs. M2).** For Y-3650 and 25747, the shared-parameter model M1 produced poor fits for specific treatments, motivating the treatment-specific parameterization M2. We present the results for both models to document the necessity of treatment-specific competition coefficients.

**Fig. 8:**
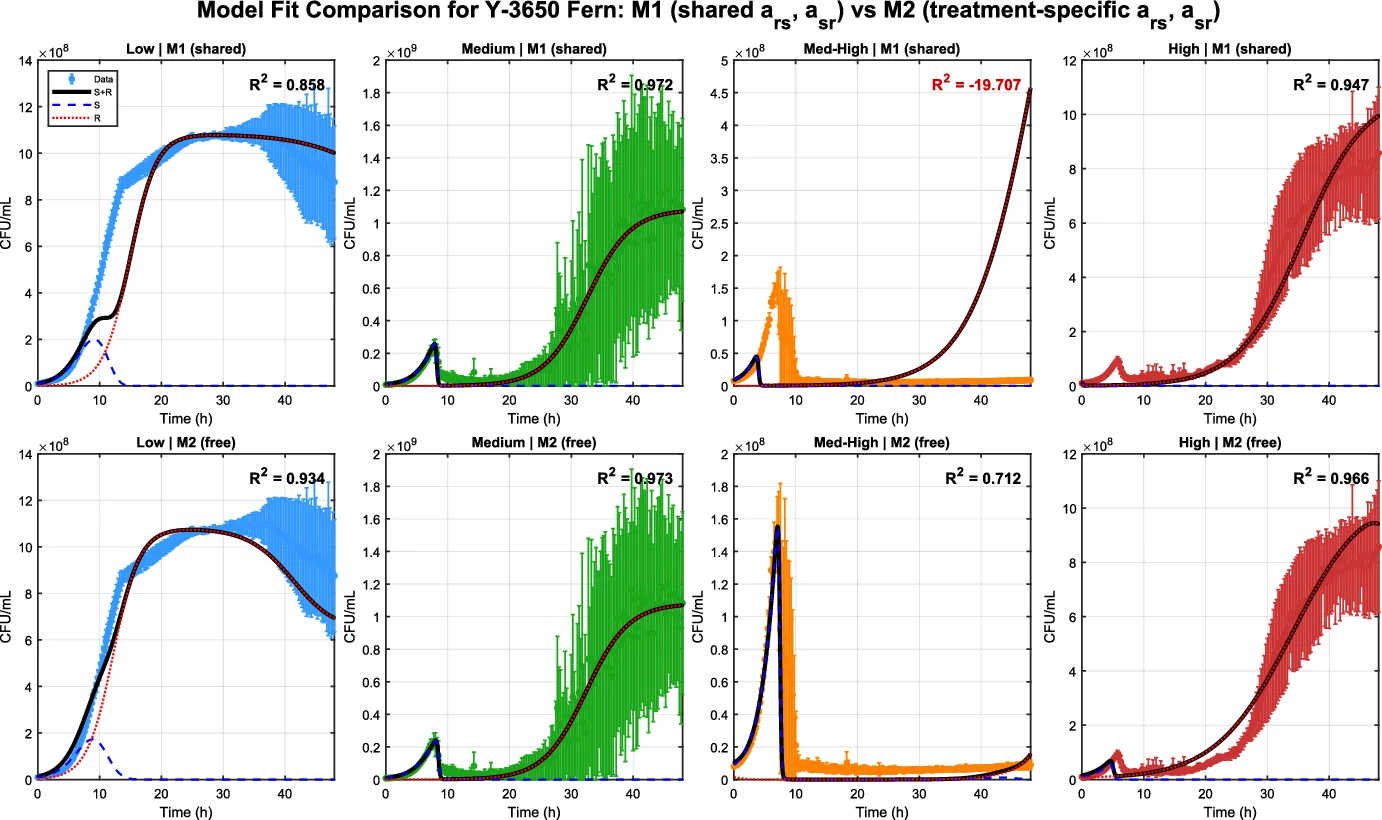
Comparison of model fits for *P. larvae* strain Y-3650 challenged with phage Fern IDv1 under two parameterizations. The top row shows fits under model M1 with shared interspecific competition coefficients across treatments, and the bottom row shows fits under model M2 with treatment-specific interspecific competition coefficients. Columns correspond to the Low, Medium, Med-High, and High treatments.(color figure online)

Under M1, Y-3650 exhibited a catastrophic failure in the Med-High treatment ($R^2=-19.71$), with acceptable fits in the remaining three treatments (Figures 8, top row; Table 4). The negative $R^2$ indicates that the model prediction was worse than a horizontal line at the treatment mean—a clear signal that the shared competition coefficients are incompatible with the dynamics at this treatment level. Notably, the Med-High treatment produced complete suppression of bacterial growth, yet the High treatment—with a greater initial phage dose—resulted in two-phase growth with eventual resistant outgrowth. This non-monotonic response cannot be captured by varying only the initial phage density $P_0$ while holding all ecological parameters fixed. For 25747, M1 similarly failed in specific treatments: the Medium treatment yielded $R^2=0.44$, and the High treatment yielded $R^2=-0.12$ (Figures 10, top row; Table 5).

**Fig. 9:**
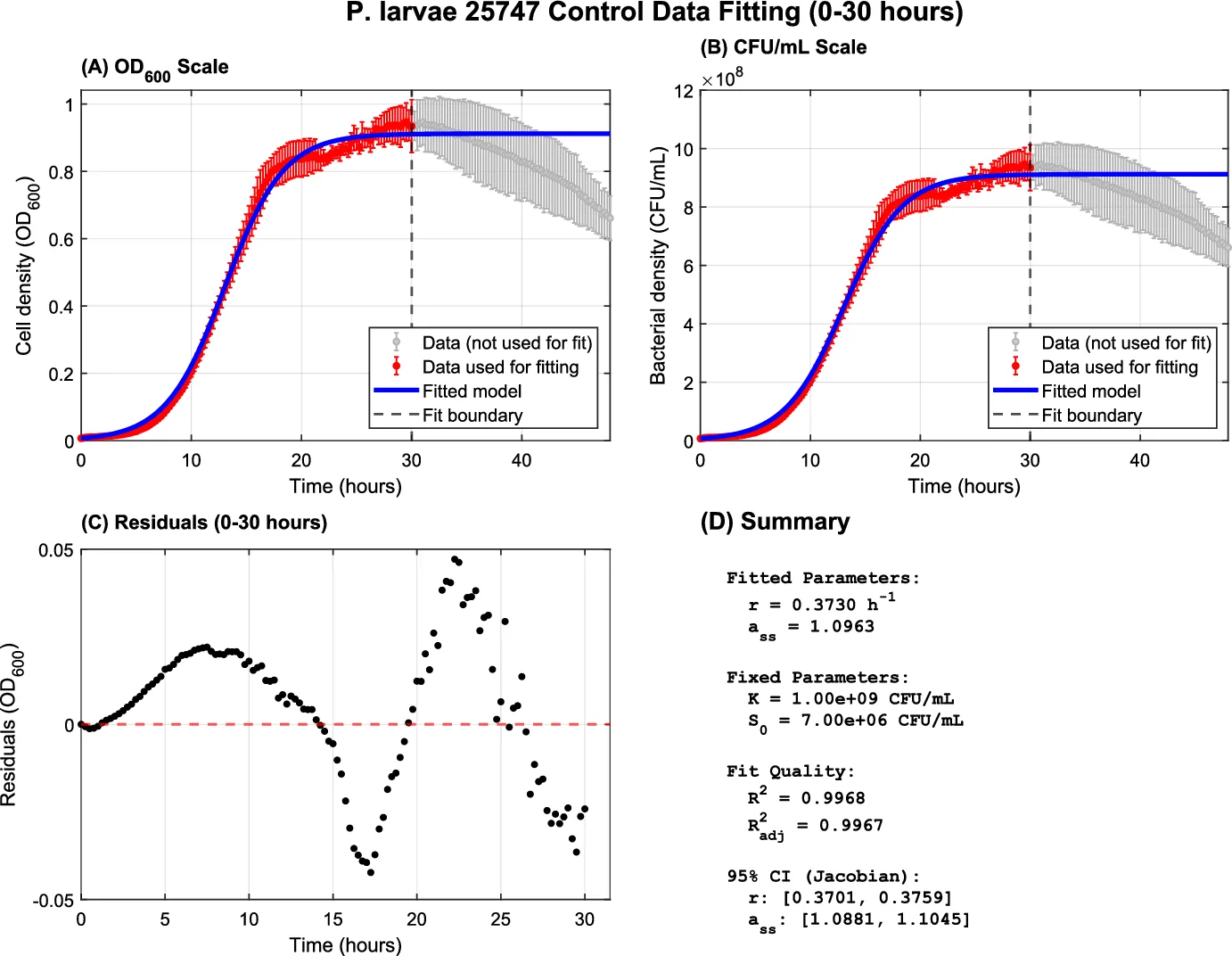
Control fit for *P. larvae* strain 25747 in the absence of phage. Panel (A) shows the $\text {OD}_{600}$ data and the fitted logistic growth curve over the 0–30 h fitting window. Panel (B) shows the corresponding fit on the CFU/mL scale. Panel (C) presents the residuals, and Panel (D) reports the estimated control parameters and goodness-of-fit statistics.(color figure online)

**Fig. 10:**
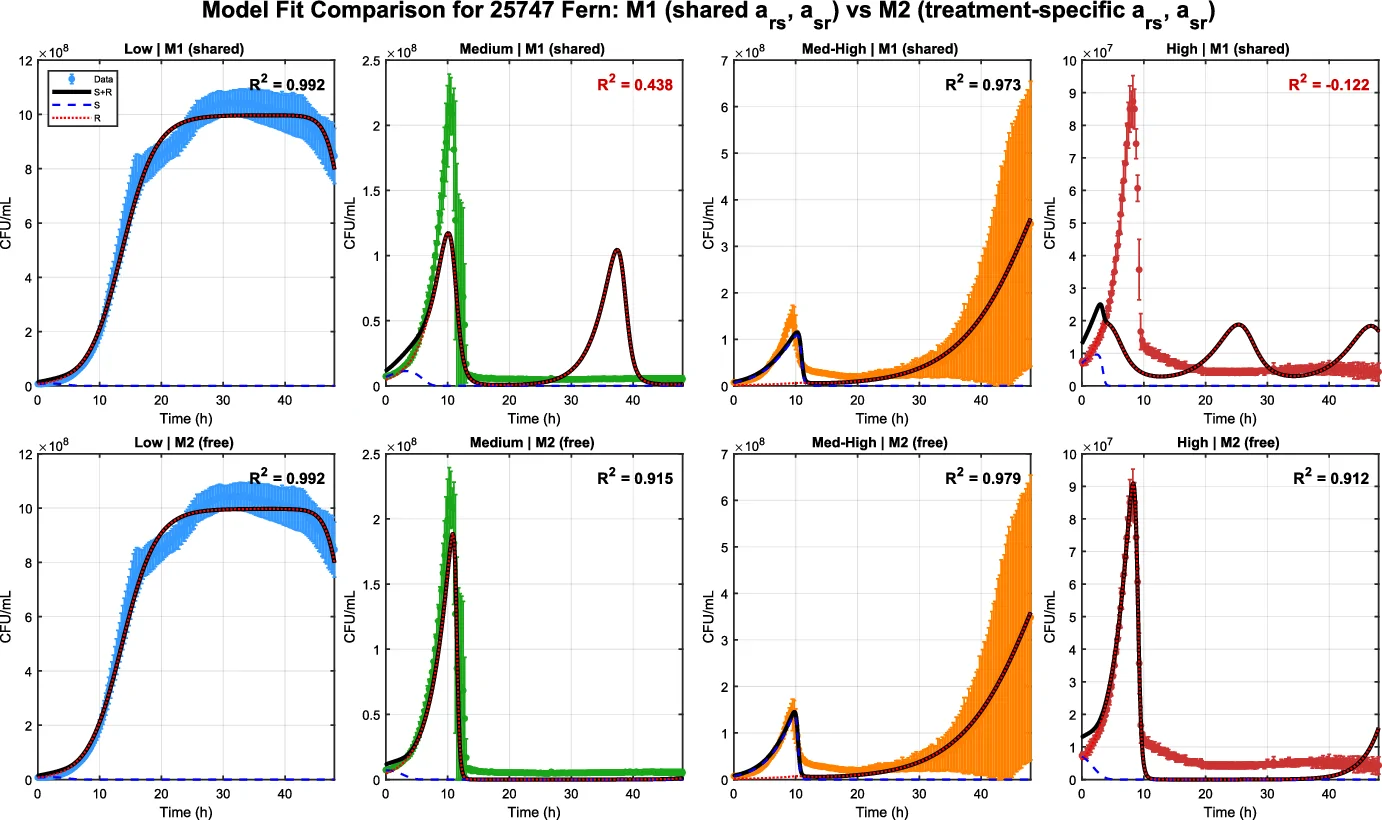
Comparison of model fits for *P. larvae* strain 25747 challenged with phage Fern IDv1 under two parameterizations. The top row shows fits under model M1 with shared interspecific competition coefficients across treatments, and the bottom row shows fits under model M2 with treatment-specific interspecific competition coefficients. Columns correspond to the Low, Medium, Med-High, and High treatments.(color figure online)

Under M2—where $a_{rs}$ and $a_{sr}$ are allowed to vary across treatments while $a_{rr}$, β, and *m* remain shared—fit quality improved substantially for both strains (Figures 8 and 10, bottom rows). For Y-3650, M2 achieved per-treatment $R^2$ values of 0.934 (Low), 0.973 (Medium), 0.712 (Med-High), and 0.966 (High), with a mean of 0.896. The most dramatic improvement occurred in the Med-High treatment, which increased from $R^2=-19.71$ under M1 to $R^2=0.712$ under M2. For 25747, M2 achieved per-treatment $R^2$ values of 0.992 (Low), 0.915 (Medium), 0.979 (Med-High), and 0.912 (High), with a mean of 0.95. The Medium treatment improved from $R^2=0.44$ to $R^2=0.92$, and the High treatment improved from $R^2=-0.12$ to $R^2=0.91$.

**Information-theoretic model comparison.** Formal comparison of M1 and M2 using the Akaike Information Criterion (AIC) and Bayesian Information Criterion (BIC) provided decisive support for M2 in both *P. larvae* strains. For Y-3650, $\Delta \text {AIC}=\text {AIC}_{\text {M2}}-\text {AIC}_{\text {M1}}=-753$ and $\Delta \text {BIC}=\text {BIC}_{\text {M2}}-\text {BIC}_{\text {M1}}=-725$ (Table 4). For 25747, $\Delta \text {AIC} = -458$ and $\Delta \text {BIC} = -430$ (Table 5). Both values far exceeded the conventional threshold of $|\Delta \text {AIC}|>10$ for decisive evidence in favor of the more complex model (Burnham and Anderson 2002). The BIC, which penalizes model complexity more heavily than the AIC, likewise favors M2, indicating that the improvement in fit is not an artifact of the additional parameters.

**Biological Interpretation.** The contrast between the *P. aeruginosa* and *P. larvae* fitting results reveals that phage pressure alters competitive dynamics between susceptible and resistant bacteria in a strain- and dose-dependent manner. For the two *P. aeruginosa* strains, a single pair of interspecific competition coefficients sufficed across all MOI levels, suggesting that MOI does not alter the competitive landscape for these bacteria. For the two *P. larvae* strains, the effective competition coefficients varied substantially with MOI, indicating that the net competition interaction between susceptible and resistant subpopulations depends on the phage environment. This dependence is inconsistent with apparent competition mediated by the phage (Holt 1977): at different phage densities, the balance of phage-mediated mortality, nutrient release from lysed cells, and selection for different resistance mechanisms shifts the effective competitive landscape.

The fitted parameter values in Tables 4 and 5 provide further insight. In Y-3650 under M2, $a_{rs}$ ranged from 4.5 (Low) to 271.1 (Med-High), indicating the competitive pressure of resistant bacteria on susceptible bacteria increased dramatically at intermediate phage doses. Similarly, $a_{sr}$ ranged from 0.03 (Low) to 232.8 (Med-High), indicating a reciprocal intensification. In 25747 under M2, $a_{rs}$ ranged from 33.1 (Med-High) to 223.1 (High), while $a_{sr}$ ranged from 0.03 (Med-High) to 2.34 (Low). The treatment-specific patterns differed between the two strains, consistent with strain-specific differences in resistance mechanisms and their associated fitness costs.

These results imply that phage resistance evolution is fundamentally shaped by ecological competition. The fact that M1—which assumes competition is independent of phage doses—fails for *P. larvae* but succeeds for *P. aeruginosa* demonstrates that the degree to which phage pressure modifies competitive interaction is itself a biologically variable feature that depends on the phage–host system.

### Profile likelihood analysis

The model comparison in Section 3.1 demonstrated that treatment-specific interspecific competition coefficients ($a_{rs}$ and $a_{sr}$) are required for both *P. larvae* strains. To provide a more direct assessment of whether these parameters can be constrained to shared values, we performed a profile likelihood analysis for each coefficient in each strain.

**Methodology.** For each strain, we fixed $a_{rs}$ (or $a_{sr}$) at each of 40 log-spaced values spanning the range of treatment-specific estimates, and re-optimized all remaining parameters independently for each treatment by nonlinear least squares. This procedure yields a profile $R^2$ curve for each treatment as a function of the fixed parameter value. If a shared value were appropriate, the per-treatment profile curves would peak at the same location; divergent optima indicate that the treatments require fundamentally different coefficient values.

**Fig. 11:**
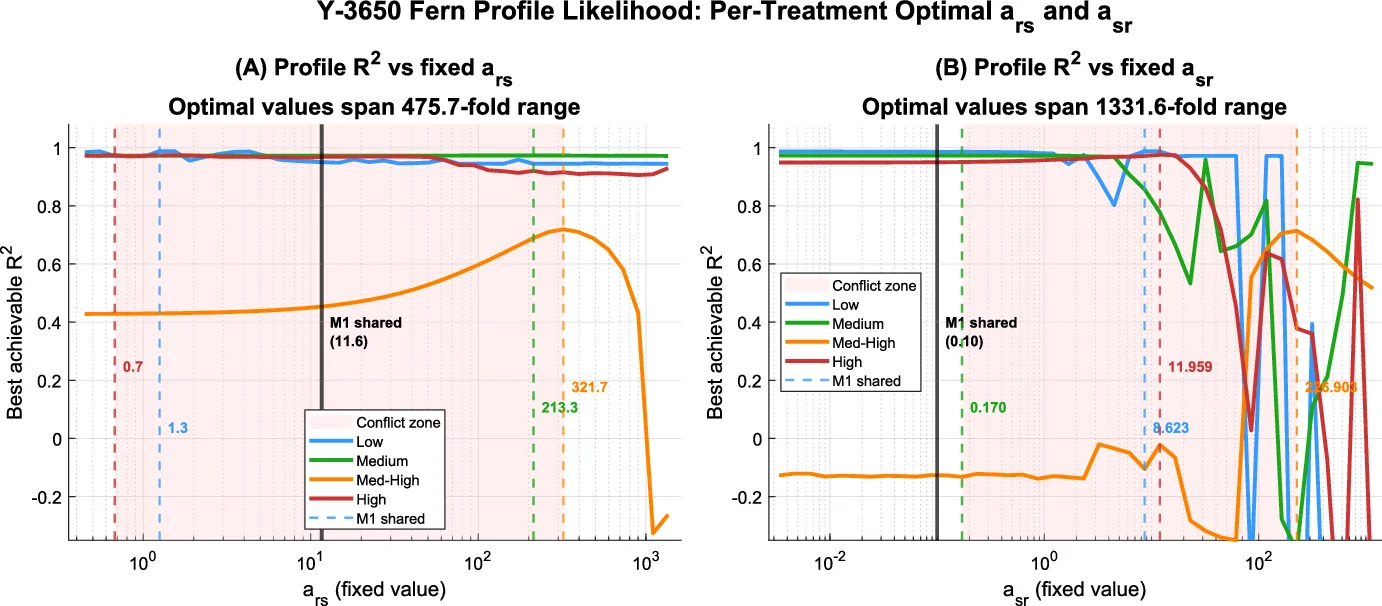
Profile likelihood analysis of interspecific competition parameters for *P. larvae* Y-3650 challenged with phage Fern IDv1. Each curve shows the best achievable $R^2$ for a given treatment when the indicated parameter is fixed at the value on the horizontal axis and all remaining parameters are re-optimized. Panel (A): profiles for $a_{rs}$ (effect of resistant bacteria on susceptible growth). Per-treatment optima (colored dashed lines) span a 476-fold range, from 0.7 (High) to 321.7 (Med-High). The M1 shared value ($a_{rs} = 11.6$, black vertical line) lies far from the Med-High optimum, incurring a substantial $R^2$ penalty. Panel (B): profiles for $a_{sr}$ (effect of susceptible bacteria on resistant growth). Per-treatment optima span a 1332-fold range, from 0.170 (Medium) to 226.9 (Med-High). The M1 shared value ($a_{sr} = 0.10$) drives the Med-High $R^2$ below zero (cost of 0.840 $R^2$ units), explaining the catastrophic M1 failure for this treatment. The pink shaded region (conflict zone) spans the range of per-treatment optima, illustrating the impossibility of selecting a single shared value that simultaneously serves all treatments (Color figure online).

**Y-3650 results.** The profile $R^2$ curves for $a_{rs}$ revealed that per-treatment optimal values span a 476-fold range, from 0.7 (High) to 321.7 (Med-High) (Figure 11, Panel A). For $a_{sr}$, the per-treatment optima span a 1332-fold range, from 0.17 (Medium) to 226.9 (Med-High) (Figure 11, Panel B). The M1 shared values ($a_{rs}=11.6$, $a_{sr}=0.1$) fall far from the treatment-specific optima for the Med-High treatment. Constraining $a_{rs}$ to its M1 shared value costs 0.265 $R^2$ units for the Med-High treatment. Constraining $a_{sr}$ to its M1 shared value drives the Med-High $R^2$ below zero (cost of 0.84 $R^2$ units), explaining the catastrophic M1 failure for this treatment.

**Fig. 12:**
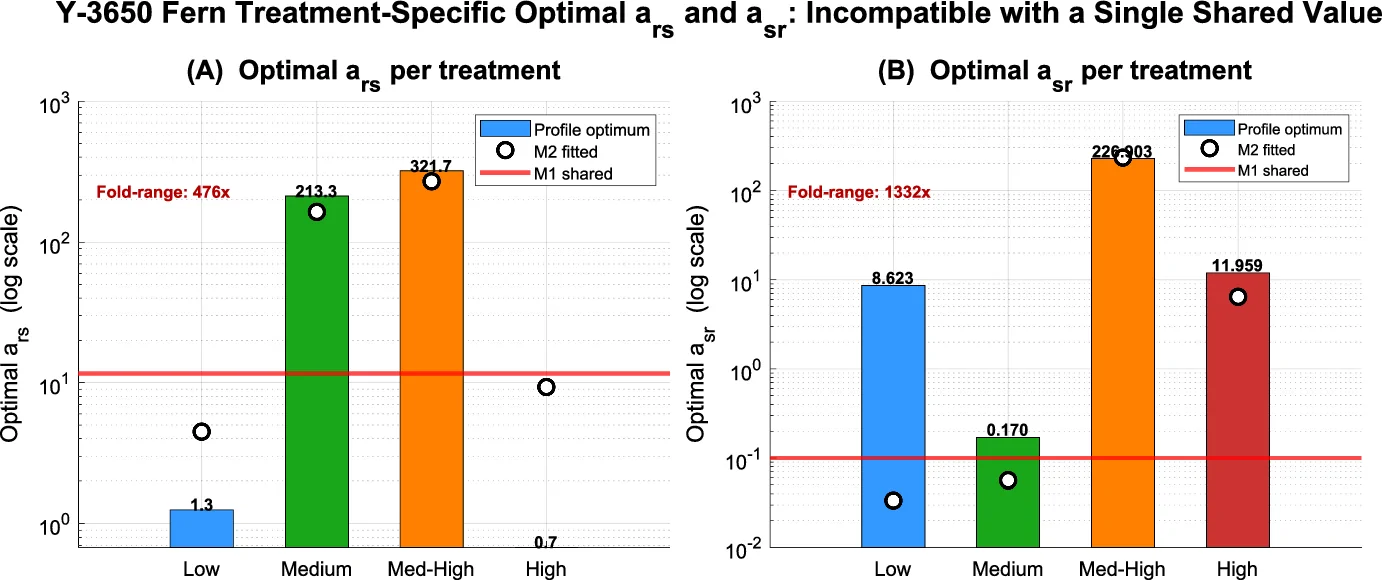
Per-treatment optimal values of interspecific competition parameters for *P. larvae* Y-3650 challenged with phage Fern IDv1, derived from the profile likelihood analysis in Figure 11. Bar heights indicate the profile-derived optimal values on a log scale; open circles show the corresponding M2 fitted values; red horizontal lines mark the M1 shared values. Panel (A): optimal $a_{rs}$ values. The Med-High treatment requires a value roughly 460-fold larger than High, and the M1 shared value ($a_{rs} = 11.6$) substantially underestimates the Med-High and Medium optima while overestimating the Low and High optima. Panel (B): optimal $a_{sr}$ values. The Med-High treatment requires $a_{sr} \approx 227$, more than three orders of magnitude larger than Medium ($a_{sr} \approx 0.17$). In both panels, the M1 shared values represent poor compromises that cannot adequately serve any individual treatment, confirming that the interspecific competition coefficients are fundamentally treatment-specific.(color figure online)

The extreme divergence in the Med-High treatment coincides with its non-monotonic dose–response: this treatment produced complete suppression of bacterial growth, whereas the High treatment—with more phage—yielded two-phase growth. This non-monotonic dose–response requires very different effective competition coefficients at intermediate versus high phage doses, a feature that the shared parameterization cannot accommodate (Figure 12). We caution that the Med-High and Medium groups are also among the treatments most affected by the cell-clumping–induced $\text {OD}_{600}$ variability noted in the Methods (Figure 2D); consequently, the extreme fitted value of $a_{sr}$ for the Med-High treatment may partly reflect distortion of the mean growth trajectory rather than a purely biological signal, and the present data cannot fully separate these contributions.

**Fig. 13:**
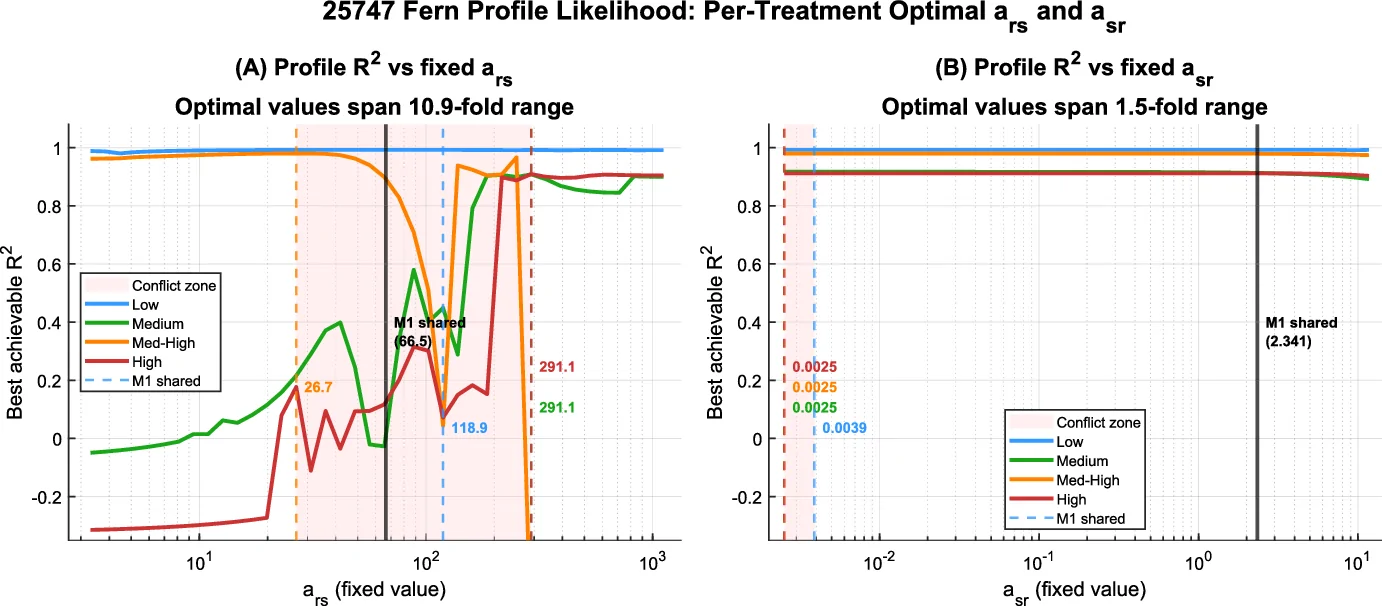
Profile likelihood analysis of interspecific competition parameters for *P. larvae* strain 25747 challenged with phage Fern IDv1, following the same methodology as Figure 11. Panel (A): profiles for $a_{rs}$. The Medium and High treatments prefer large values ($a_{rs} \approx 291$), while Med-High prefers a much smaller value ($a_{rs} = 26.7$), yielding a 10.9-fold range of optima. The M1 shared value ($a_{rs} = 66.5$) falls within the conflict zone but costs up to 0.936 $R^2$ units for the Medium treatment. Panel (B): profiles for $a_{sr}$. All four treatments converge on very small optimal values ($a_{sr} \approx 0.0025$–0.0039), but the M1 shared value ($a_{sr} = 2.341$) overshoots by nearly three orders of magnitude. This inflation is a signature of compensatory parameter adjustment: when $a_{rs}$ is constrained to a suboptimal shared value under M1, the optimizer inflates $a_{sr}$ to partially offset the misspecification.(color figure online)

**Fig. 14:**
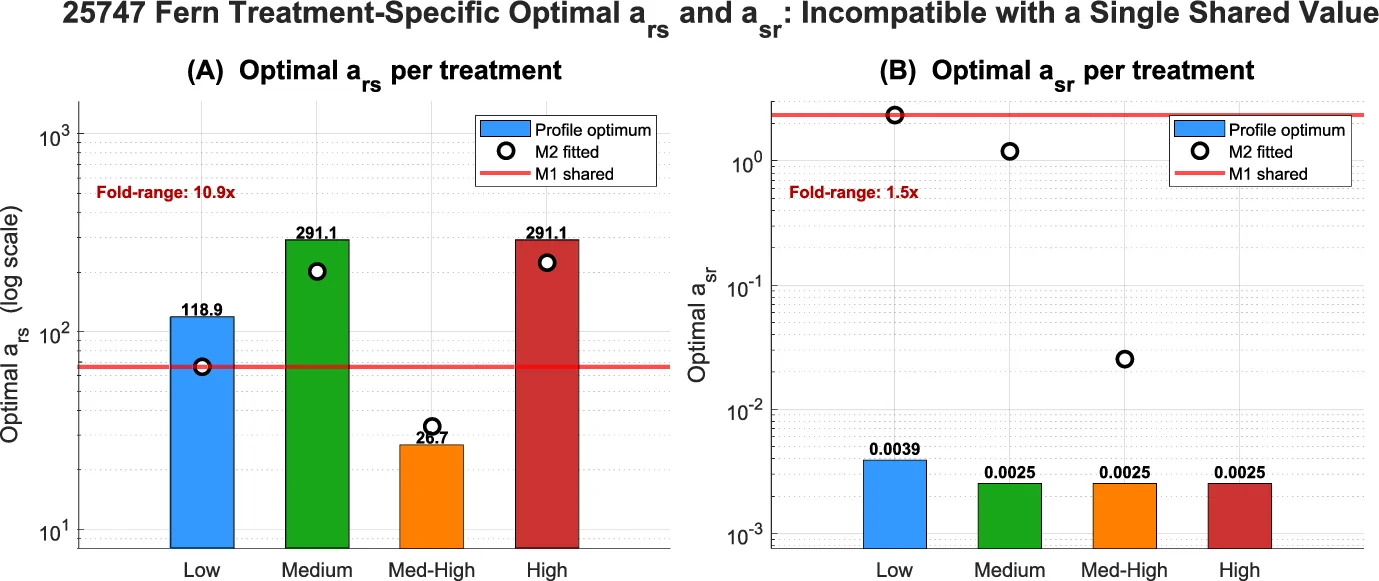
Per-treatment optimal values of interspecific competition parameters for *P. larvae* strain 25747 challenged with phage Fern IDv1, derived from the profile likelihood analysis in Figure 13. Panel (A): optimal $a_{rs}$ values exhibit a bimodal pattern in which Medium and High treatments cluster around $a_{rs} \approx 291$ while Med-High requires a much smaller value ($a_{rs} = 26.7$). The M1 shared value ($a_{rs} = 66.5$, red line) compromises between these groups but fits neither well. Panel (B): optimal $a_{sr}$ values are uniformly small across all treatments (≈0.0025), yet the M1 shared value ($a_{sr} = 2.341$) exceeds them by roughly 1000-fold. The close agreement between profile-derived optima (bars) and M2 fitted values (open circles) for $a_{rs}$ validates the M2 parameterization, while the large discrepancy between the M1 shared $a_{sr}$ and all treatment-specific optima confirms that the M1 constraint produces biologically implausible parameter estimates.(color figure online)

**25747 results.** For $a_{rs}$, per-treatment optimal values span a 10.9-fold range, from 26.7 (Med-High) to 291.1 (Medium and High) (Figure 13, Panel A). The M1 shared value ($a_{rs} = 66.5$) costs up to 0.936 $R^2$ units for the Medium treatment. For $a_{sr}$, all treatments prefer very small values (0.0025–0.0039), but the M1 shared value ($a_{sr} = 2.34$) is approximately 1000-fold higher (Figure 13, Panel B). This inflation of the M1 shared $a_{sr}$ is a signature of compensatory parameter adjustment: when $a_{rs}$ is constrained to a suboptimal shared value, the optimizer inflates $a_{sr}$ to partially offset the misspecification, resulting in biologically implausible parameter estimates (Figure 14).

**Cross-strain synthesis.** Despite quantitative differences in the magnitude of parameter divergence, both *P. larvae* strains exhibit the same qualitative pattern: the interspecific competition coefficients vary systematically with MOI in ways that no single shared value can accommodate. For Y-3650, optimal $a_{rs}$ values span a 476-fold range and optimal $a_{sr}$ values span a 1332-fold range. For 25747, the corresponding ranges are 10.9-fold and 1.5-fold. The convergence of three independent lines of evidence—profile likelihood analysis showing conflicting optima, information-theoretic model comparison decisively favoring treatment-specific parameterization ($\Delta \text {AIC}=-753$ for Y-3650; $\Delta \text {AIC}=-458$ for 25747), and substantial $R^2$ improvement under M2—across two genetically distinct *P. larvae* strains establishes that MOI-dependent competition dynamics are a general feature of the phage–bacteria interaction, not a strain-specific artifact.

## Discussion

This study combined a generalized Lotka–Volterra model of phage–bacteria dynamics with experimental growth data from four bacterial strains to investigate how the multiplicity of infection shapes resistance evolution. Two principal findings emerged. First, the interspecific competition coefficients $a_{rs}$ and $a_{sr}$ are MOI-dependent in *P. larvae*, as confirmed by both information-theoretic model comparison and profile likelihood analysis. Second, the competitive context created by phage pressure—rather than the intrinsic mutation rate—is the primary determinant of resistance evolution outcomes. We discuss the implications of these findings for ecological theory, phage therapy, and future modeling efforts.

**Phage-mediated competition drives MOI-dependent dynamics.** In the generalized Lotka–Volterra framework used here, $a_{rs}$ and $a_{sr}$ are effective competition coefficients that subsume all net interactions between susceptible and resistant bacteria, including direct resource competition, phage-mediated indirect competition, and nutrient release from phage-lysed cells. Because these indirect effects are themselves phage-density dependent, but not accounted for via an interaction term with *P*, the effective coefficients may vary with MOI when the model does not explicitly resolve the underlying mechanistic pathways. Indeed, even phage parameters traditionally considered intrinsic—such as burst size—are known to depend on host physiological state: Nabergoj et al. (2018) demonstrated that burst size of phage T4 on *E. coli* varies over a 10-fold range (from 8 to 89 PFU· $\text {cell}^{-1}$) as a function of bacterial growth rate in continuous culture. Because different MOI treatments alter the growth dynamics and resource environment of the host population, variation in effective phage and competition parameters across treatments reflects genuine biological heterogeneity rather than a fitting artifact, although measurement noise from cell clumping may contribute to the most extreme estimates, as discussed in the Experimental limitations. The profile likelihood analysis (Section 3.2) confirmed this expectation empirically: for both *P. larvae* strains, per-treatment optima for $a_{rs}$ and $a_{sr}$ diverged by up to three orders of magnitude, and no single shared value could simultaneously serve all four treatment levels (Figures 11–14).

The MOI-dependence of the effective competition coefficients reflects a composite of mechanisms that our phenomenological model cannot disentangle. In Holt’s (1977) apparent competition framework (Holt 1977), two prey sharing a common predator interact indirectly through shared predation pressure even when direct interspecific competition coefficients are zero. In our system, by contrast, the phage *P* is modeled explicitly, yet $a_{rs}$ and $a_{sr}$ are demonstrably nonzero and vary by orders of magnitude with MOI. The influence of phage pressure on the competitive landscape therefore represents an effect over and above the shared-predator interaction: for the *P. larvae* strains, even a description that combines direct and apparent competition under a single shared parameterization fails to capture the data (Tables 4 and 5), implying that the phage reshapes the competition itself in a density-dependent manner. Several mechanisms could produce this MOI-dependence. First, phage-mediated lysis of susceptible cells releases nutrients into the local environment; at higher MOI, this release is more rapid and may selectively benefit resistant bacteria – creating a competitive advantage that scales with phage density but is not captured by a shared-predator indirect effect alone. Second, different MOI treatments select for qualitatively different resistance mechanisms with distinct fitness costs and competitive phenotypes (discussed above in the context of γ and ϵ), so that the effective competition landscape shifts not only because phage density changes but because the composition of the resistant subpopulation itself changes. Third, MOI-dependent phage evolution (Singhal and Turner 2021) may alter phage host-range and adsorption characteristics over the 48-hour window, further reshaping the effective interaction between subpopulations. Future models could represent these mechanisms explicitly—for example, by including nutrient dynamics as a state variable, by introducing phage-density-dependent competition terms $a_{rs}(P)$ and $a_{sr}(P)$, or by tracking multiple resistance classes arising under different MOI regimes.

The need for treatment-specific competition coefficients is also evident from the qualitative structure of the data. If the interspecific competition coefficients and the other ecological parameters were identical across treatments, the model would predict a monotonic relationship between phage dose and suppression severity, because only the initial condition $P_0$ would differ between treatments. The observed non-monotonic patterns—in which a higher phage dose can paradoxically yield weaker suppression—demonstrate that the effective competitive interactions between susceptible and resistant bacteria shift as a function of phage pressure. These qualitative inversions cannot be reproduced by varying only $P_0$ while holding all ecological parameters fixed, consistent with our model comparison and profile likelihood analyses (Section 3.2).

The dependence of the competition parameters on phage pressure is consistent with arms-race dynamics in phage–bacteria coevolution. Buckling and Rainey (2002) demonstrated that bacterial resistance and phage infectivity coevolve reciprocally, with the nature and cost of resistance mechanisms changing in response to phage selection pressure. Weitz et al. (2005) showed theoretically that phage–bacteria coevolutionary dynamics produce qualitatively different ecological outcomes depending on the strength of selection, and that the effective fitness landscape of resistant bacteria depends on the intensity of phage predation. In our experiments, each MOI treatment imposes a different selective regime over the 48-hour observation window, which can select for different resistance mechanisms or different levels of resistance, thereby altering the effective values of ϵ, γ, and ϕ. Higher phage pressure is expected to select for stronger (and potentially costlier) resistance mechanisms, while lower phage pressure may permit weaker, less costly resistance to predominate.

**Connection to the density-dependent framework for phage therapy.** Payne and Jansen (2001) established a foundational theoretical framework for understanding phage therapy as a density-dependent kinetic process. Their analysis identified several qualitatively distinct outcomes depending on the initial ratio of phage to bacteria, including successful bacterial clearance, failed therapy due to insufficient phage amplification, and intermediate regimes of partial suppression. The three growth patterns we observed experimentally—delayed growth, two-phase growth, and complete suppression (Figure 2)—correspond to analogous regimes in the Payne–Jansen framework, with two-phase growth representing an intermediate outcome where initial phage amplification suppresses susceptible bacteria but resistant variants ultimately emerge. Our contribution extends this framework by demonstrating that the boundaries between these regimes are not determined solely by phage-to-bacteria ratios and lysis kinetics, but also by the competitive dynamics between susceptible and resistant subpopulations. The non-monotonic dose–response patterns observed in both *P. larvae* strains—where an intermediate phage dose produced complete suppression while a higher dose permitted resistant outgrowth—cannot be explained by the standard density-dependent model without incorporating MOI-dependent competition.

**Implications for phage therapy.** The non-monotonic dose–response patterns observed in *P. larvae* Y-3650 and 25747 carry a practical implication: higher phage doses do not invariably produce better therapeutic outcomes. In Y-3650, the Med-High treatment produced complete suppression while the High treatment—with ten-fold more phage—permitted two-phase growth and eventual resistant outgrowth (Figure 2D). This observation suggests that, at very high phage densities, the rapid depletion of susceptible bacteria may reduce phage-mediated indirect pressure on resistant cells, paradoxically facilitating their proliferation. If this mechanism operates in vivo, then optimizing phage dosing for therapeutic applications requires consideration not only of direct bacteria killing but also of the competitive environment that the treatment creates for resistant subpopulations (Levin and Bull 2004; Payne and Jansen 2003).

Our results suggest that resistant management in phage therapy may be more effectively achieved through ecological manipulation—controlling the competitive conditions that determine whether resistant subpopulations can proliferate—than through approaches aimed at suppressing mutation rates. This perspective aligns with the broader recognition that phage resistance is often mediated by trade-offs between resistance and other fitness components (León and Bastías 2015; Burmeister et al. 2020), and that the fitness cost of resistance is context-dependent (Meaden et al. 2015). In our model, the fitness cost γ and the competitive coefficients jointly determine whether resistant bacteria can sustain growth; even a modest fitness cost can prevent resistant outgrowth when competitive pressures are sufficiently strong. We note, however, that these implications are derived from in vitro experiments with simplified two-population dynamics, and validation in more complex in vivo setting will be necessary before translating these findings into dosing guidelines.

**Experimental limitations.** Several experimental limitations should be noted when interpreting our results. First, our model treats all resistant bacteria as a single subpopulation *R*, but in practice, multiple resistance mechanisms may coexist within a culture, each with distinct fitness costs and competitive properties (Labrie et al. 2010; Wright et al. 2018). The MOI-dependence of $a_{rs}$ and $a_{sr}$ may partially reflect shifts in the composition of the resistant subpopulation—different MOI treatments selecting for different resistance mechanisms—rather than changes in the competitive ability of a single resistant type. Direct characterization of the resistance mechanisms arising at different MOI levels would help resolve this question.

Second, our experiments did not track phage evolution during the 48-hour assays. Phage counter-adaptation, including host-range mutations that partially overcome bacterial resistance, can occur on timescales relevant to our experiments (Buckling and Rainey 2002; Weitz et al. 2005). Moreover, MOI itself can shape phage evolutionary strategies: Singhal and Turner (2021) demonstrated that bacteriophage populations evolved under low MOI develop greater host exploitation ability, while those evolved under high MOI develop intracellular competitive traits, indicating that phage life-history characteristics are not invariant across MOI conditions. If such phage evolution occurred, it would be absorbed into the effective parameters of our phenomenological model, potentially contributing to the observed MOI-dependence of the competition coefficients.

Third, the “resistant” subpopulation in our model is operationally defined by growth in the presence of phage, but we did not independently verify whether this growth reflects genetically encoded resistance, phenotypic tolerance, or persister cell dynamics (Attrill et al. 2021; Turkington et al. 2019). These mechanisms differ in their heritability and fitness consequences, and distinguishing among them would require additional experimental characterization. Mutations that affect cell clumping could explain the observation of highly variable OD 600 readings (Figure 2D), but we did not confirm this in this study. It is important to distinguish two separate features of the medium and medium-high treatments. The first is a measurement effect: cell clumping renders the $\text {OD}_{600}$ signal stochastic, and because these groups are among the noisiest, the extreme interspecific competition coefficient $a_{sr}$ fitted for the Med-High treatment of Y-3650 may be inflated by distortion of the mean growth trajectory rather than reflecting a purely biological interaction. Averaging over replicates and time points reduces, but does not remove, this source of error. The second is the non-monotonic dose–response itself—complete suppression at the Med-High dose but resistant outgrowth at the higher High dose—which is a genuine dynamical feature that the present model can fit only with treatment-specific competition coefficients and cannot yet explain mechanistically; we are investigating its biological basis, including the associated genetic changes, in ongoing follow-up work. In a previous study (Spencer et al. 2025), we characterized mutations conferring resistance to phages and found several that could affect cell growth. However, this study was performed on solid media. Characterizing the growth of these strains in liquid culture would be interesting.

**Model limitations and future directions.** The adequacy of M1 for the *P. aeruginosa* strains but not for the *P. larvae* strains warrants careful interpretation. The PA103 Rd3 and PAK Rd3 datasets each comprise only 9 time points over 18 hours, compared to 193 time points over 48 hours for each *P. larvae* treatment group. This difference in data density means that the *P. aeruginosa* datasets may lack the statistical power to distinguish M1 from M2: M1’s adequacy for these strains could reflect insufficient temporal resolution rather than the genuine biological constancy of the competition coefficients. Collecting denser time-series data for *P. aeruginosa*—ideally with continuous optical density monitoring as used for *P. larvae*—would allow a more definitive test of whether competition coefficients are truly MOI-dependent in these strains.

Our model makes several simplifying assumptions that future work could relax. First, the model aggregates all resistant bacteria into a single compartment *R*. A natural extension would introduce multiple resistance classes—for example, first-order resistant bacteria $R_1$ that are resistant to the original phage $P_1$ but susceptible to a host-range mutant phage $P_2$, and second-order resistant bacteria $R_{12}$ that are resistant to both the original $P_1$ and a host-range mutant phage $P_2$. Such a model could test whether the MOI-dependence of $a_{rs}$ and $a_{sr}$ arises from compositional shifts within the resistant subpopulation, a hypothesis that cannot be evaluated with the current single *R* formulation. Second, our model assumes instantaneous lysis upon phage adsorption. Incorporating an explicit latent period through delay differential equations may improve the fit to early-time dynamics and would allow investigation of how latent period duration interacts with MOI to influence resistance evolution (Levin and Bull 2004; Cairns et al. 2009).

Third, the deterministic ODE framework cannot capture the stochastic fluctuations that may be important at low population densities—for example, during the initial emergence of resistant mutants or at the onset of phage amplification. Stochastic differential equation (SDE) models of the Itô type offer a natural extension that preserves the continuous-time structure of our ODEs while incorporating demographic and environmental noise. The application of Itô SDEs to phage–bacteria systems remains limited. Carletti (2004) introduced stochastic perturbations into a marine bacteriophage infection model and numerically simulated the resulting Itô system, and subsequently established mean-square stability conditions for a stochastic phage model with time delay (Carletti 2007). Bardina et al. (2013) proved concentration results for a delayed SDE model of phage therapy, showing that in the small-noise regime the system converges to a bacteria-free equilibrium with high probability. The most comprehensive framework to date is that of Vidurupola and Allen (2014), who derived both continuous-time Markov chain (CTMC) and Itô SDE models for phage–bacteria dynamics applicable to phage therapy, and its extension by Vidurupola et al. (2018), who incorporated resistant bacteria, bacteriophage complexes, and bacterial debris into the Itô SDE framework. However, none of these stochastic models incorporate the competitive dynamics between susceptible and resistant subpopulations that are central to our findings. Extending our generalized Lotka–Volterra model to an Itô SDE framework would allow investigation of how stochastic fluctuations interact with MOI-dependent competitive dynamics—for instance, whether noise at low phage doses can stochastically trigger transitions between the delayed growth and two-phase growth regimes, or whether the probability of resistant outgrowth depends on the competitive environment in ways not captured by deterministic models.

Finally, extending the model to structured environments such as biofilms, where spatial heterogeneity can alter both phage predation dynamics and competitive interactions (Chegini et al. 2020; Bisht et al. 2021), represents an important step towards ecological realism.

**Concluding remarks.** This study demonstrates that phage resistance evolution is fundamentally an ecological process in which the competitive context—shaped by phage pressure—determines population-level outcomes independently of intrinsic mutation capacity. The profile likelihood analysis provides direct empirical evidence that the effective interspecific competition coefficients between susceptible and resistant bacteria are MOI-dependent, an effect that extends beyond phage-mediated apparent competition and reflects a direct, phage-density-dependent reshaping of the competitive interaction between susceptible and resistant bacteria. The non-monotonic dose–response patterns observed in *P. larvae* reveal that the relationship between phage dose and resistance evolution is more complex than previously appreciated, with important implications for the rational design of phage therapy protocols. By bridging mathematical modeling with experimental microbiology, our work contributes to a growing understanding that resistance evolution must be managed as an ecological problem, not merely a genetic one.

## Data Availability

All experimental data analyzed and simulation MATLAB codes used to produce all the figures in this study are available at https://github.com/tphan86/BPI_model
